# Identifying corals displaying aberrant behavior in Fiji’s Lau Archipelago

**DOI:** 10.1371/journal.pone.0177267

**Published:** 2017-05-24

**Authors:** Anderson B. Mayfield, Chii-Shiarng Chen, Alexandra C. Dempsey

**Affiliations:** 1 National Museum of Marine Biology and Aquarium, Checheng, Pingtung, Taiwan; 2 Khaled bin Sultan Living Oceans Foundation, Annapolis, MD, United States of America; 3 Taiwan Coral Research Center, Checheng, Pingtung, Taiwan; 4 Graduate Institute of Marine Biotechnology, National Dong Hwa University, Checheng, Pingtung, Taiwan; 5 Department of Marine Biotechnology and Resources, National Sun Yat-Sen University, Kaohsiung, Taiwan; King Abdullah University of Science and Technology, SAUDI ARABIA

## Abstract

Given the numerous threats against Earth’s coral reefs, there is an urgent need to develop means of assessing reef coral health on a proactive timescale. Molecular biomarkers may prove useful in this endeavor because their expression should theoretically undergo changes prior to visible signs of health decline, such as the breakdown of the coral-dinoflagellate (genus *Symbiodinium*) endosymbiosis. Herein 13 molecular- and physiological-scale biomarkers spanning both eukaryotic compartments of the anthozoan-*Symbiodinium* mutualism were assessed across 70 pocilloporid coral colonies sampled from reefs of Fiji’s easternmost province, Lau. Eleven colonies were identified as outliers upon employment of a detection method based partially on the Mahalanobis distance; these corals were hypothesized to have been displaying aberrant sub-cellular behavior with respect to their gene expression signatures, as they were characterized not only by lower *Symbiodinium* densities, but also by higher levels of expression of several stress-targeted genes. Although these findings could suggest that the sampled colonies were physiologically compromised at the time of sampling, further studies are warranted to state conclusively whether these 11 scleractinian coral colonies are more stress-prone than nearby conspecifics that demonstrated statistically normal phenotypes.

## Introduction

Earth’s coral reefs are currently threatened by a number of anthropogenic insults [1–2], most notably global climate change [3–4]. There is consequently an urgent need to develop means of assessing coral health on a proactive timescale [5]. Unfortunately, traditional coral reef surveys (e.g., [6]) involve the documentation of dead or dying corals; although the ensuing data are indeed of interest to managers, they come too late to benefit the resident corals. Ideally, an assessment of coral health could be made prior to visible manifestations of stress, such as bleaching, whereby the coral-dinoflagellate (genus *Symbiodinium*) endosymbiosis that serves as the foundation of all coral reefs deteriorates [7].

Molecular biology-based approaches have shed light on numerous aspects of the fundamental and stress biology of anthozoan-dinoflagellate endosymbioses [9–12], and molecular biomarkers [8], in particular, may hold promise for coral health diagnostics since they require only a single sampling event; therefore, they could theoretically be used to make inferences about coral physiology prior to visible signs of stress, such as bleaching. A particular stress protein, for instance, may demonstrate up-regulation in response to an environmental shift well before any loss of *Symbiodinium* from the coral gastrodermal tissues. If a significant proportion of a reef’s corals are expressing highly abnormal levels of well-validated biomarkers, then it is conceivable that a manager could be alerted to attempt to ameliorate the impact of the local-scale stressors (e.g., water pollution) in order to promote coral resilience.

To validate a potential biomarker, such as an mRNA, one would ideally collect data from control specimens to establish a “normal” concentration level. However, even if traditional tank studies are employed (e.g., [13]), what is considered a control expression level of a biomarker in one region may be aberrant in another. It should be noted here that “aberrant” does not refer to health, but only to divergence from a norm/average. Unfortunately, the fact that no reefs on Earth are devoid of any human impact precludes the ability to simply present typical ranges for each marker (above or below-which signifies stress) in the absence of data from corals sampled prior to the Industrial Revolution. As potential evidence for this, high expression levels of stress genes have been measured in corals from some of the most remote, least populated regions of the Pacific Ocean, such as the Austral and Cook Islands [14]. This phenomenon was hypothesized previously [15–16] to represent mRNA “front-loading,” whereby high expression levels of mRNAs encoding stress proteins (e.g., heat shock proteins [HSPs][17]) occur at all times in order for the corals to have the capacity to rapidly translate such stress proteins when temperatures change abruptly due to, for instance, upwelling [18–19]. However, corals of the Austral and Cook Islands experience relatively low and stable temperatures [20], suggesting that this may not *only* be a strategy employed by corals residing within thermally extreme and dynamic environments (such as those of Southern Taiwan [15]). It is worth noting that corals are amongst the only organisms currently known to exhibit such an “always stressed” phenotype given the significant cellular energy expenditure required to do so [21].

**Fig 1:**
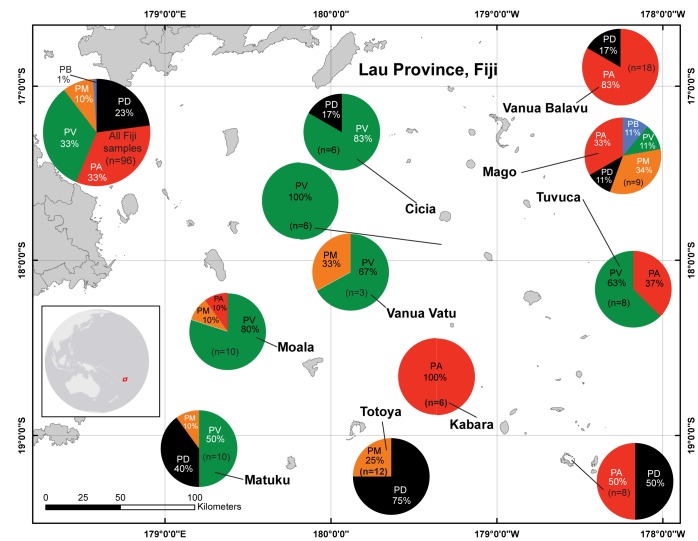
Map of Fiji’s Lau Province and pie graphs depicting the proportional genetic breakdown of the pocilloporids sampled. PA = *P*. *acuta* (red; Sebastian Schmidt-Roach [SSR; taxonomic authority] genotype β). PB = *P*. *brevicornis* (blue). PD = *P*. *damicornis* (black; SSR genotype α). PM = *P*. *meandrina* (orange). PV = *P*. *verrucosa* (green). The color codes for the five species are used throughout all of the manuscript’s figures. All images of the sampled colonies (including “macro” images of the polyps) can be found on the following website: http://coralreefdiagnostics.com under the “Fiji” sub-heading.

**Fig 2:**
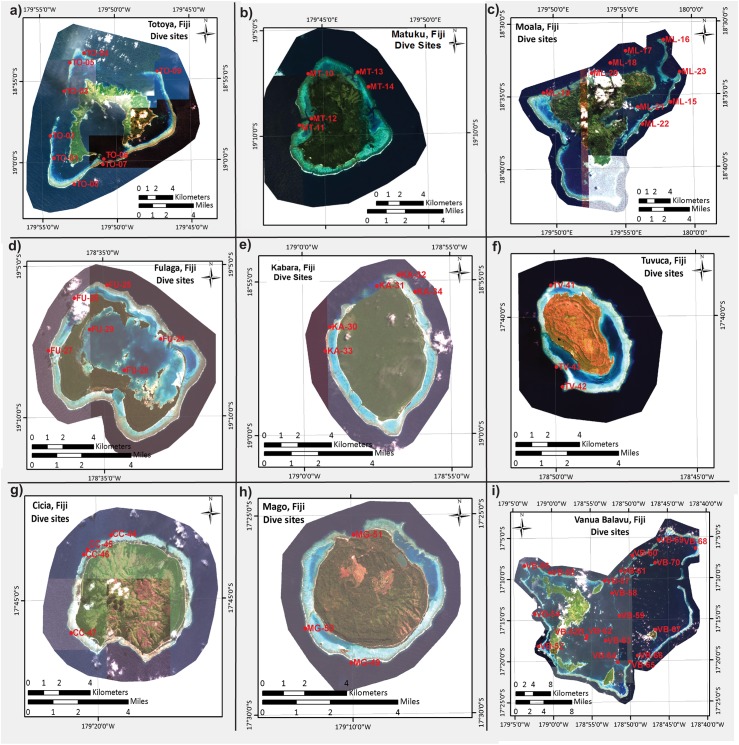
Maps of nine of the islands/atolls within Fiji’s Lau Archipelago whose reefs were surveyed. Two islands, Vanua Vatu (June 15, 2013) and Nayau (June 16, 2013), have not been depicted, as the corals sampled were not processed in full for all response variables. For a detailed description of the reefs of the former, including high-resolution maps, please see Saul and Purkis [26]. Site 53 (Vanua Balavu) has not been labeled due to its proximity to site 56 (see GPS coordinates in Table 1.).

Despite issues with using absolute expression levels of individual genes or proteins to predict whether or not a coral is stressed, it is possible that multivariate statistical approaches (MSA) could nevertheless be used to identify corals behaving significantly differently from what is normal in a particular region. Colonies displaying statistically unusual phenotypes may ultimately be found to be those either experiencing stress or, in contrast, those of enhanced resilience (assuming the front-loading hypothesis to be true). To test the notion that molecular biomarkers could be used to identify aberrantly behaving coral colonies, the model coral *Pocillopora damicornis* [22–25] was targeted across reefs of Fiji’s frontier province, Lau (Figs 1 and 2), and 13 molecular-physiological response variables were measured in each colony. MSA were used to analyze the dataset and identify outliers, and it was hypothesized that certain environmental parameters might significantly influence outlier frequency; for instance, it was predicted that corals displaying statistically aberrant behavior would be more likely to be found on reefs with higher temperatures and light levels. Collectively, it was hoped that this MSA-based approach for assessing the environmental physiology of this model reef coral could serve as a conceptual platform for others looking to use, in particular, molecular biology-driven approaches for not only identifying outliers, but also for simply establishing baseline functional data for invertebrate-dinoflagellate endosymbioses in understudied regions of the Indo-Pacific.

## Materials and methods

### Field surveys and sample collection

In June 2013, the Khaled bin Sultan Living Oceans Foundation’s research vessel, the *M*.*Y*. *Golden Shadow*, traversed a significant portion of Fiji’s Lau Archipelago (Fig 1) upon invitation from the Fijian government. For comparative purposes, surveys would ideally have also been conducted in more impacted areas, such as near the population centers of Suva. However, the government expressed a sincere need for research in Lau, the most remote and least studied region of the country; therefore, field work was limited to this archipelago. Remote sensing, field surveys, and sample collection were undertaken as described previously [20], and certain details have been reiterated in the Methods A in S1 File. Briefly, sites were chosen by analysis of satellite data in conjunction with visual observations from seaplane flyovers, and the western and northern sides of the islands were generally prioritized given the high seas and strong winds characteristic of the southern and eastern sides at the time of surveying. Benthic maps were created, and surveys were conducted (see the Methods A in S1 File.) to document the dominant benthic organisms and substrate types. Eleven islands/atolls were visited (Table 1 and Fig 2), with typically 1–2 days spent diving around each. In total, 70 sites were surveyed over approximately one month (Table 1).

**Table 1:** Site information. The average temperatures (temp.), salinities, and live coral cover (ALCC) percentages for each island reflect the means across the sites from which corals were sampled only (n = 46 reef sites), and the environmental parameters highlighted in **bold** differed significantly between islands (1-way analysis of variance [ANOVA], *p*<0.05). Letters behind standard deviations represent Tukey’s honestly significant difference groups (*p*<0.05) between island means for temp. and ALCC. For the environmental data for all 70 surveyed sites, please see the S1 Data File. ND = not determined. PA = *P*. *acuta*. PB = *P*. *brevicornis*. PD = *P*. *damicornis*. PM = *P*. *meandrina*. PV = *P*. *verrucosa*.

ISLAND Site	Exposure	Reef type	Reef zone	Latitude	Longitude	Date (2013)	Temp. (°C)	Salinity (unit-less)	ALCC (%)	#corals analyzed/ #collected (sample ID #)	*Pocillopora* spp. present
**TOTOYA** (sampled 20 corals across 7 of the 9 sites surveyed)											
FJTO02	intermediate	barrier	fore reef	-18.9728	-179.9068	Jun. 3	27.2	35.3	36.2	2/2 (#4–5)	PD only
FJTO03	intermediate	barrier	fore reef	-18.9273	-179.8907	Jun. 3	27.2	35.2	34.2	1/1 (#6)	PD only
FJTO04	exposed	barrier	fore reef	-18.8886	-179.8677	Jun. 4	27.0	35.3	25.0	0/1	ND
FJTO05	intermediate	barrier	fore reef	-18.8981	-179.8836	Jun. 4	27.1	35.3	45.6	3/3 (#2–3, 7)	PD only
FJTO06	protected	patch	lagoon	-18.9976	-179.8473	Jun. 4	27.3	35.3	37.8	2/5 (#9–10)	PD only*
FJTO07	intermediate	barrier	fore reef	-19.0032	-179.8485	Jun. 5	27.2	35.2	41.8	1/3 (#15)	PD only*
FJTO08	intermediate	barrier	fore reef	-19.0230	-179.8808	Jun. 5	27.4	35.6	36.0	4/5 (#16–17, 19–20)	PM & PD*
				**Totoya avg.±std. dev.**			27.2**±**0.1^a^	35.3±0.1	36.7±6.5^ab^	13/20	
**MATUKU** (sampled 10 corals across 3 of the 5 sites surveyed)											
FJMT10	intermediate	barrier	fore reef	-19.1178	179.7382	Jun. 6	26.9	34.8	41.6	3/3 (#21–23)	PV only
FJMT13	exposed	barrier	fore reef	-19.1172	179.7783	Jun. 7	26.8	35.2	27.7	6/6 (#24–29)	PD, PV, & PM
FJMT14	exposed	barrier	fore reef	-19.1290	179.7866	Jun. 7	26.8	35.2	31.8	1/1 (#30)	PD only
				**Matuku avg.±std. dev.**			26.8±0.1^abe^	35.1±0.2	33.7±7.1^ab^	10/10	
**MOALA** (sampled 17 corals across 6 of the 9 sites surveyed)											
FJML16	intermediate	barrier	fore reef	-18.5204	179.9656	Jun. 8	27.2	35.3	33.0	0/1	ND
FJML17	intermediate	barrier	fore reef	-18.5325	179.9200	Jun. 8	27.2	35.3	32.8	3/5 (#33–35)	PV & PM*
FJML18	intermediate	barrier	fore reef	-18.5461	179.9013	Jun. 9	27.1	35.3	40.4	5/5 (#37–41)	PV only
FJML19	intermediate	barrier	fore reef	-18.5794	179.8201	Jun. 9	27.2	34.8	51.0	1/2 (#42)	PV only*
FJML20	intermediate	barrier	fore reef	-18.5577	179.8785	Jun. 9	27.2	35.3	36.8	1/1 (#44)	PA only
FJML21	protected	patch	back reef	-18.5972	179.9337	Jun. 10	26.9	35.2	21.6	2/3 (#45, 47)	PV only*
					**Moala avg.±std. dev.**		27.1**±**0.1^ab^	35.2±0.2	35.9±9.7^ab^	12/17	
**FULAGA** (sampled 9 corals across 4 of the 6 sites surveyed)											
FJFL24	protected	patch	back reef	-19.1240	-178.5480	Jun. 11	26.7	35.5	42.0	1/1 (#48)	PD only
FJFL25	intermediate	barrier	fore reef	-19.0940	-178.5809	Jun. 11	26.5	35.2	44.7	3/4 (#49–51)	PA & PD*
FJFL27	exposed	barrier	fore reef	-19.1299	-178.6174	Jun. 12	26.5	35.2	42.8	0/1	ND
FJFL29	protected	patch	lagoon	-19.1184	-178.5918	Jun. 12	26.5	35.2	50.3	3/3 (#54–56)	PA only
					**Fulaga avg.±std. dev.**		26.6±0.1^c^	35.3**±**0.2	45.0±3.7^a^	7/9	
**KABARA** (sampled 13 corals across 2 of the 5 sites surveyed)											
FJKB31	protected	pinnacles	lagoon	-18.9194	-178.9577	Jun. 13	27.2	35.4	38.5	4/10 (#60, 66, 68–69)	PA only*
FJKB34	exposed	barrier	fore reef	-18.9228	-178.9363	Jun. 14	26.5	35.3	22.3	0/3	ND
				**Kabara avg.±std. dev.**			26.9±0.5^abc^	35.4**±**0.1	30.4**±**11^ab^	4/13	
**VANUA VATU** (sampled 6 colonies across the 3 sites surveyed)											
FJVV35	exposed	barrier	fore reef	-18.3864	-179.2786	Jun. 15	26.7	35.3	48.8	1/1 (#70)	PV only
FJVV36	intermediate	barrier	fore reef	-18.3438	-179.2803	Jun. 15	26.6	35.3	32.8	1/4 (#71)	PM only*
FJVV37	intermediate	barrier	back reef	-18.3583	-179.2847	Jun. 15	26.7	35.2	41.8	1/1 (#75)	PD only
				**Vanua Vatu avg.±std. dev.**			26.7±0.1^bc^	35.3±0.1	41.1±8.0^ab^	3/6	
**NAYAU** (sampled 7 colonies across 1 of the 3 sites surveyed)											
FJNA38	intermediate	barrier	fore reef	-17.9512	-179.0670	Jun. 16	27.0^abc^	35.6	48.0^ab^	6/7 (#76–77, 79–82)	PD & PV*
**TUVUCA** (sampled 8 colonies across 2 of the 3 sites surveyed)											
FJTV41	intermediate	barrier	fore reef	-17.6498	-178.8354	Jun. 17	26.8	34.7	28.2	6/6 (#83–88)	PA & PV
FJTV42	intermediate	barrier	fore reef	-17.7041	-178.8291	Jun. 17	27.0	35.4	36.8	2/2 (#89–90)	PA only
				**Tuvuca avg.±std. dev.**			26.9±0.1^ab^	35.1±0.5	32.5±6.1^ab^	8/8	
**CICIA** (sampled 12 colonies across 3 of the 5 sites surveyed)											
FJCC44	intermediate	barrier	fore reef	-17.7167	-179.3243	Jun. 18	27.1	35.5	42.2	6/9 (#91, 93, 95, 97- 99)	PV only*
FJCC47	exposed	barrier	fore reef	-17.7671	-179.3491	Jun. 19	27.1	35.5	40.7	1/2 (#101)	PD only*
FJCC48	intermediate	barrier	fore reef	-17.7498	-179.3841	Jun. 19	26.2	34.4	46.5	1/1 (#102)	PV only
				**Cicia avg.±std. dev.**			26.8±0.5^abc^	35.1±0.6	43.1±3.0^ab^	8/12	
**MAGO** (sampled 9 colonies across the 3 sites surveyed)											
FJMG49	exposed	Barrier	fore reef	-17.4785	-179.1672	Jun. 20	27.1	35.5	53.4	1/1 (#103)	PB only
FJMG50	intermediate	Barrier	fore reef	-17.4639	-179.1877	Jun. 20	27.1	35.5	41.7	5/5 (#104–108)	PV, PD, & PM
FJMG51	intermediate	fringing	fore reef	-17.4249	-179.1655	Jun. 20	27.4	35.4	47.0	3/3 (#109–111)	PA only
					**Mago avg.±std. dev.**		27.2±0.2^ab^	35.5±0.1	47.4±5.9^a^	9/9	
**VANUA BALAVU** (sampled 42 colonies across 12 of the 19 sites surveyed											
FJVB52	intermediate	barrier	fore reef	-17.3028	-179.0309	Jun. 21	27.2	35.5	47.4	4/4 (#112–115)	PD & PA
FJVB53	intermediate	barrier	fore reef	-17.1394	-179.0600	Jun. 21	27.3	35.5	29.0	3/3 (#116–118)	PA only
FJVB55	intermediate	barrier	fore reef	-17.1534	-179.0049	Jun. 22	27.1	35.4	48.8	4/6 (#119–120, 123- 124)	PA only*
FJVB56	intermediate	barrier	fore reef	-17.1395	-179.0599	Jun. 22	ND	ND	28.7	0/1	ND
FJVB58	protected	patch	lagoon	-17.1960	-178.8707	Jun. 22	ND	ND	10.7	0/3	ND
FJVB60	intermediate	barrier	fore reef	-17.1206	-178.8265	Jun. 23	27.0	35.4	11.0	2/5 (#130–131)	PD only*
FJVB61	intermediate	barrier	fore reef	-17.1518	-178.8512	Jun. 23	27.1	35.6	14.5	1/1 (#134)	PA only
FJVB62	protected	patch	lagoon	-17.2824	-178.9267	Jun. 23	27.0	35.4	33.0	1/4 (#138)	PA only*
FJVB63	protected	patch	lagoon	-17.2923	-178.8856	Jun. 24	26.8	35.5	28.7	2/10 (#146–147)	PA only*
FJVB65	protected	barrier	back reef	-17.3355	-178.8337	Jun. 25	26.8	35.5	4.33	0/2	ND
FJVB66	protected	patch	lagoon	-17.3234	-178.8167	Jun. 25	26.6	35.3	11.8	1/2 (#151)	PA only*
FJVB67	protected	fringing	lagoon	-17.2709	-178.7774	Jun. 25	26.8	35.4	21.5	1/1 (#153)	PA only
				**Vanua Balavu avg.±std. dev.**			27.0±0.2^abc^	35.5±0.1	24.1±14^b^	19/42	
				**Lau Archipelago avg.±std. dev.**			27.0±0.3	35.3±0.2	35.0±12		
		Sampled 153 colonies across 46 of the 70 sites surveyed									
				Total # analyzed for molecular-scale response variables/total # genotyped						70/96	

*Certain samples at site were not genotyped, so other pocilloporid species may have been present.

Pocilloporid corals (n = 153) were sampled from 46 of the 70 surveyed reefs (Table 1). The target was the α genotype of *P*. *damicornis* [27], though when it was not present, morphologically similar congenerics, such as *P*. *acuta* (genotype β), were instead sampled as described previously [20]; additional details of the sampling procedure and colony size analysis (i.e., maximum [max.] length [cm; response variable #1] and planar surface area [SA; cm^2^; response variable #2] measurements) can be found in the Methods A in S1 File. Briefly, colonies were sampled across a number of environmental gradients (e.g., temperatures, light levels, and depths; Table 1 and S1 Table) and presented an array of pigmentation states (S1 Table). Photosynthetically active radiation (PAR) was measured next to each colony at the time of sampling with an Odyssey meter (“integrating PAR sensor,” Dataflow Systems, New Zealand) that had been calibrated against a LiCor LI-193 instrument (USA) and programmed to log at 10-s intervals. The coral samples were transported from Fiji to Taiwan aboard a commercial aircraft under a permit (PVPS1300609) issued by the Fiji’s Ministry of Defence (sp.), National Security, and Immigration. Additionally, ministers from this organization were aboard the ship at the time research was being undertaken, and they approved all collections. Finally, tribal chiefs were always consulted with official government translators before SCUBA diving in their territorial waters.

### Nucleic acid extractions and molecular response variables

From 100 of the 153 pocilloporid colonies sampled, both RNAs and DNAs were extracted as described in the Methods A in S1 File, and an RNA/DNA ratio was calculated for each sample (response variable #3). Then, 96 and 70 colonies were genotyped and analyzed for the 10 remaining molecular response variables described below, respectively; the majority of the latter 70 colonies were also genotyped. From the RNAs, five and four *Symbiodinium* and host coral target genes, respectively, were targeted after conversion of RNA to cDNA (described in the Methods A in S1 File). The mRNAs spanned five cellular processes that were hypothesized to be environmentally sensitive based on our current knowledge of coral ecophysiology and the stress/bleaching response, in particular (see [4] and references therein.): photosynthesis, metabolism, cell adhesion, light modulation, and the stress response. The five *Symbiodinium* genes (S2 Table; response variables #4–8) included the photosynthesis gene ribulose-1,5-bisphosphate carboxylase/oxygenase large subunit (*rbcL*), the metabolism gene zinc-induced facilitator-like 1-like (*zifl1l*; known to be down-regulated at high temperature in *Symbiodinium* populations within *P*. *damicornis*; [25]), and three genes encoding proteins involved in the cellular stress response: *hsp90*, ubiquitin ligase (*ubiq-lig*; [28]), and ascorbate peroxidase (*apx1*). The host coral target genes (S2 Table; response variables #9–12) were the metabolism gene carbonic anhydrase (*ca*), the cell adhesion gene *lectin*, the stress gene copper-zinc superoxide dismutase (*cu-zn-sod*), and the light absorbing gene green fluorescent protein-like chromoprotein (*gfp-cp*). Corals characterized by either highly elevated or severely diminished expression levels of these genes were hypothesized to be displaying aberrant behavior at the time of sampling. The details of how such aberrancy was quantified are discussed in detail below. Real-time PCR (qPCR)-based gene expression quantification of these nine targets is described in the Methods A in S1 File.

From the DNAs co-extracted from the same biopsies from which RNAs were purified, host and *Symbiodinium* genome copy proportions (GCP) were calculated (*sensu* [29]), and the latter served as a proxy for *Symbiodinium* density in each sample (response variable #13). In addition to the recovery of an exogenous RNA spike (discussed in the Methods A in S1 File), host and *Symbiodinium* gene expression data were normalized to the host and *Symbiodinium* GCP, respectively. This controls for variable ratios of host/*Symbiodinium* between samples [30], which can vary greatly due to, for instance, bleaching. DNAs were also used to genotype the *Symbiodinium* populations in each sample to clade level using the primers and qPCR assays of Correa et al. [31]. When a threshold cycle (Ct) value <35 was documented for a particular clade assay, the sample was deemed positive for that clade. Finally, the DNA was also used to genotype the host corals via PCR amplification of a portion of the mitochondrial genome encompassing the 3’ end of the ATP synthase (subunit 6) gene and the 5’ end of the mitochondrial control region (formerly called the mitochondrial open reading frame [mORF]). The genotyping protocol and consequent sequence analysis were performed as in a prior work [20], and samples were assigned to one of five species (Fig 1).

### Overview of the statistical analyses

A variety of statistical analyses were utilized to attempt to 1) understand the relationship between environment and coral physiology, 2) identify outliers, and 3) understand the differences between outliers and statistically normally behaving colonies. Regarding the first aim, traditional, univariate analysis of variance (ANOVA) was first used to test the effects of all 14 environmental parameters (discussed below) on the 13 physiological and molecular response variables assessed (see the “Univariate statistical analyses” sub-heading.). Then, multivariate ANOVA (MANOVA) was used to determine the effects of each of the same 14 environmental parameters on the multivariate mean calculated across the 13 response variables (see the “Multivariate analysis of variance” sub-heading). Next, additional MSA were used to depict 1) variation in the dataset (principal components analysis [PCA]) and 2) similarity between samples (multidimensional scaling [MDS]; see the “Principal components analysis and multidimensional scaling” sub-heading.). It was hypothesized that these two exploratory approaches could reveal samples behaving in a statistically unusual manner from the global mean phenotype.

In addition to PCA and MDS, more quantitative means were used to identify corals displaying statistically aberrant behavior. This outlier detection method was primarily based on the Mahalanobis distance, though a univariate statistics-based approach was used to corroborate these findings (see the “Outlier determination” sub-heading.). Then, a series of both univariate and multivariate statistical tests were used to differentiate outliers from non-outliers (see the “Modeling differences between outliers and non-outliers” sub-heading.). This involved 1) direct tests of response variables between outliers and non-outliers with student’s *t*-tests and 2) a MANOVA-based canonical correlation analysis (CCA) of outliers vs. non-outliers. Finally, multivariate analysis of covariance (MANCOVA) and univariate analysis of covariance (ANCOVA) were used to test for differences in the relationships amongst response variables between the outliers and non-outliers. Unless noted otherwise, all statistical analyses were performed with JMP® (ver. 12.0.1) after confirming both normality (Shapiro-Wilk *W* test *p*>0.05) and homogeneity of variance (Levene’s test *p*>0.05) of the data. The details of all such statistical tests can be found below in the respective sections cited above in parentheses.

### Univariate statistical analyses

For the univariate statistical analyses, transformations (log or rank) were conducted when data were not normally distributed or of homogeneous variance. First, 1-way ANOVAs were used to test the influence of the following 14 environmental parameters on the 13 coral response variables: 1) island (n = 9 of the 11 islands visited; Table 1), 2) site (n = 35 reefs), 3) exposure (exposed [typically windward], protected [typically leeward or lagoonal], or intermediate [neither exposed nor protected]), 4) reef zone (fore reef, back reef, or lagoon), 5) reef type (barrier, patch, fringing, or pinnacles), 6) sampling date (n = 21 sampling days), 7) collection time (n = 3 categorical groupings: <10:00, 10:00–14:00, or >14:00), 8) collection depth (n = 7 categorical groupings: <5, 5–10, 10–15, 15–20, 20–25, 25–30, or >30 m), 9) site temperature (n = 2 categorical groupings: 26–27 vs. 27–28°C), 10) site salinity (34.7, 34.8, 34.9, 35.0, 35.1, 35.2, 35.3, 35.4, or 35.5), 11) sampling PAR (n = 4 categorical groupings: <50, 50–100, 100–200, or >200 μmol m^-2^ s^-1^), 12) average live coral cover (ALCC; n = 5 categorical groupings: 10–20, 20–30, 30–40, 40–50, or >50%), 13) host species (n = 5; see Fig 1), and 14) *Symbiodinium* assemblage (clade C only, clades A+C, or clades D+C). Although the latter two variables are not environmental parameters, they were nevertheless hypothesized to influence coral physiology.

Max. colony length, planar SA, *Symbiodinium* GCP, RNA/DNA ratio, and expression of the nine genes (n = 13 response variables) were analyzed across the 14 environmental parameters with 1-way ANOVA. Because 182 ANOVAs were performed, a Bonferroni adjustment of 13.5 was made to the *a priori*-chosen α level of 0.05, resulting in a modified α of 0.004. A similar environmental parameter x molecular physiological response variable 1-way ANOVA matrix was also generated individually for each of the species for which >15 colonies were sampled: *P*. *damicorni*s, *P*. *acuta*, and *P*. *verrucosa* (see the legend of S4 Table for details.). Host genotype frequency was also tested as a response variable, and frequency data (including outlier frequency [discussed below]) were analyzed with contingency table-based *X*^2^ tests.

### Multivariate analysis of variance

Although a Bonferroni adjustment may suffice in controlling for type I statistical errors, MSA can uncover relationships amongst response variables that are not evident from univariate-based statistics alone, while doing so at a lower false positive error rate [32]. Several MSA were taken herein to understand the relationship between environment and coral physiology. Prior to MSA, data were converted to Z-scores to control for differing scales between response variables. First, MANOVA was performed to determine the effect of each of the 14 environmental parameters on the multivariate mean (“centroid”) calculated across the 13 response variables. Briefly, MANOVA tests whether vectors of means (rather than simply individual means, which are analyzed by univariate ANOVA) of different samples are from the same distribution. Not only is it more statistically conservative than performing individual univariate ANOVAs for each dependent variable, but, when used with JMP’s “discriminant analysis” function, it can also uncover combinations of response variables that best explain differences between environmental parameters (when documented) by looking at canonical correlations.

### Principal components analysis and multi-dimensional scaling

Upon orthogonal transformation of the data into principal components (PC), PCA was performed with the 13 response variables to determine the combinations of response variables that best accounted for variation in the dataset (*sensu* [33]). A second PCA was performed with the 11 molecular-scale parameters only (i.e., excluding max. length and planar SA), as these were the response variables used to attempt to assign a level of normalcy/aberrancy to each sample (described below). As an alternate, ordination-based means of visualizing the dataset in multiple dimensions, PRIMER (ver. 5) was used to construct a Bray-Curtis similarity matrix, and an MDS plot based off of this matrix was then created. In such an MDS plot, the spatial proximity of the samples is directly proportional to their similarity (i.e., widely separated samples are relatively less similar to each other than samples adjacent to each other in the plot.). PRIMER’s analysis of similarity (ANOSIM) function was used to test for the effects of the 14 environmental parameters on separation of samples within the dataspace generated. For a more detailed explanation of MANOVA+PCA and MDS+ANOSIM, readers are referred to a classic biometry text [34] and Clarke and Warwick [35], respectively.

### Outlier determination

Several methods were used to attempt to find outliers in the dataset. First, Mahalanobis distances were calculated using the 11 molecular-scale response variables only; briefly, size was not predicted to influence sub-cellular physiology in a predictable way (i.e., larger colonies are not necessarily healthier or more normal than smaller ones.). The Mahalanobis distance is essentially the separation between a sample’s multivariate centroid and the global mean centroid. Samples characterized by distance values above the upper control limit (UCL) of 4.29 calculated by JMP were considered to be “Mahalanobis distance outliers.” Then, a heat map was generated by JMP, and samples with Z-scores <-2 or >2 for a certain response variable were given a score of 1. For instance, if a sample had a *Z*-score of -3 for one response variable and 6 for another, it would be given a “heat map score” of 2. Only when a sample’s Mahalanobis distance was >4.29 and its heat map score was ≥1 was it considered to be an outlier.

### Modeling differences between outliers and non-outliers

Several measures were taken to understand the response variables that contributed most to a sample being deemed an outlier. First, a student’s *t*-test was used to test for outlier vs. non-outlier differences for each response variable. Then, JMP’s predictor screening function was used to rank the 11 molecular response variables in order of their contribution to the cumulative difference between outliers and all other samples. Next, the *Z*-score for each response variable was regressed against the respective Mahalanobis distance for each sample, and the significance of the correlation was tested with a linear regression *t*-test. It was hypothesized that those response variables with the highest proportional contribution to the outlier vs. non-outlier difference would also demonstrate the strongest positive correlation with the Mahalanobis distance.

CCA was then performed using JMP’s discriminant analysis function to determine the response variables that best separated outliers from non-outliers along canonical axis (CA) 1. The canonical scores from this analysis were regressed against the respective Mahalanobis distances to understand the degree of congruency between these two statistics. CA1 scores were hypothesized to correlate positively with the Mahalanobis distance. Finally, MANCOVA and univariate ANCOVA were used to determine whether the relationship between biological composition (*Symbiodinium* GCP and RNA/DNA ratio) and gene expression was statistically similar between outliers and non-outliers. An α level of 0.05 was set for all MSA except for the environmental parameter vs. response variable MANOVAs (in which the α levels were Bonferroni-adjusted in a similar manner as were the univariate ANOVAs).

## Results

### Overview of the dataset

**Fig 3:**
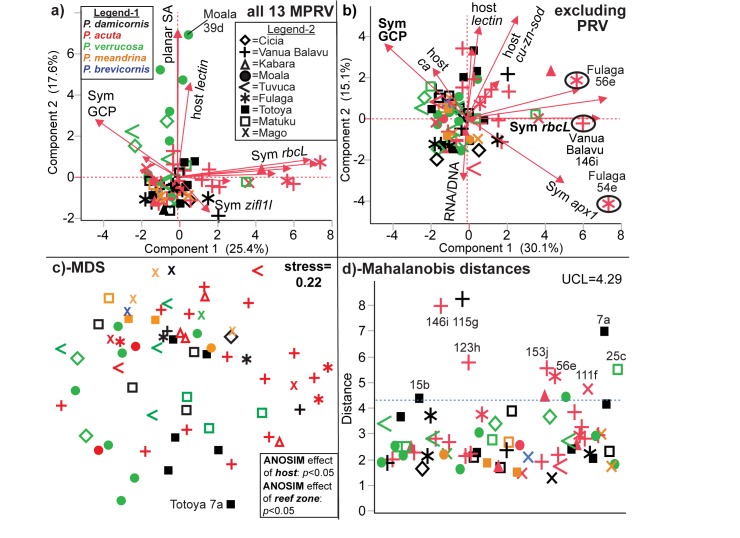
Multivariate statistical analysis of the Lau Archipelago dataset. In the principal components analysis (PCA) biplot of all 13 molecular physiological response variables (MPRV; [a]), not all axes have been labeled due to spatial constraints, and the maximum (max.) length vector falls beneath the planar surface area (SA) one. One outlier, Moala 39, has been labeled in (a). The species color codes and island symbols in the inset legends of (a) apply to all other panels. PCA was also conducted upon the exclusion of the two physiological response variables (PRV; max. length and planar SA; [b]). The three samples with the highest PC1 scores have been encircled and labeled for emphasis, and not all axes have been labeled due to spatial constraints. All PCA data (eigenvalues and eigenvectors) can be found in the S1 Data File. A multidimensional scaling (MDS) plot based on the Bray-Curtis similarity matrix has also been depicted (c), and the most separated, distinct data point (Totoya 7) has been labeled. Finally, a Mahalanobis distance plot has been presented (d), and 9 of the 11 outliers, all of which fell above the upper control limit (UCL) of 4.29 (blue, dotted, horizontal line), have been labeled; the remaining 2 were left unlabeled due to spatial constraints in the figure but fall below samples 153 and 56. These samples were all associated with heat maps scores ≥1. The following letters behind the outlier sample identification numbers denote the site of collection (Table 1): a = FJTO05, b = FJTO07, c = FJMT13, d = FJML18, e = FJFL29, f = FJMG51, g = FJVB52, h = FJVB55, i = FJVB63, and j = FJVB67. ANOSIM = analysis of similarity.

**Fig 4:**
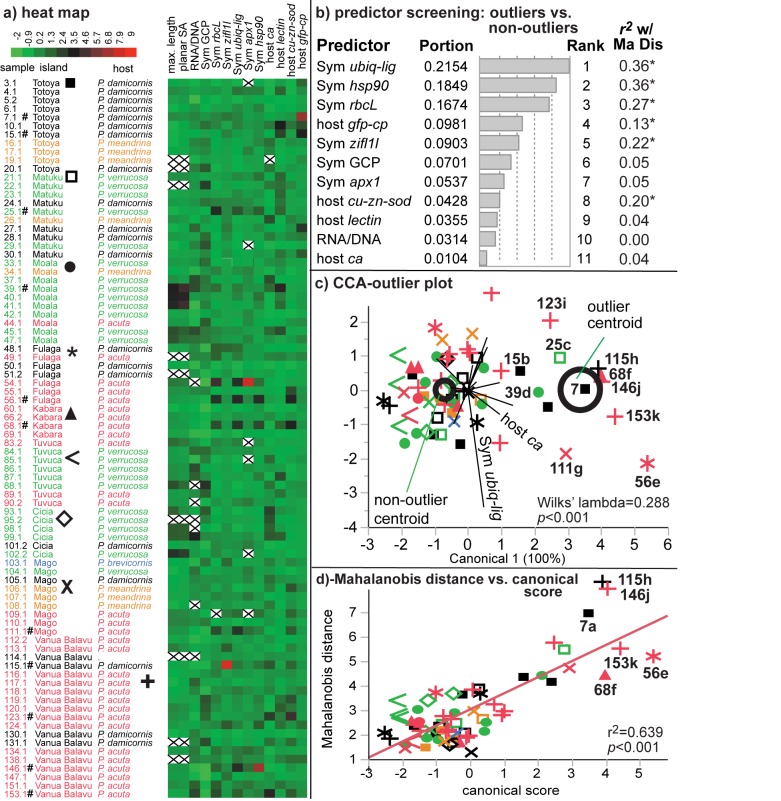
Outlier analysis of the of Lau Archipelago dataset. A heat map (a) of *Z*-scores was first used to depict variation in the dataset, and “x’s” denote missing data. Samples marked by “#” were deemed Mahalanobis distance outliers (Table 3). The symbols adjacent to island names in (a) correspond to the island symbols in (c)-(d), as do the species colors. JMP’s predictor screening function was used to rank the response variables that contributed most significantly to the cumulative difference between the 11 outliers and the 59 remaining samples (b). Then, the Z-score for each sample was plotted against its Mahalanobis distance (“Ma Dis”) for each of the 11 response variables, and statistically significant correlations (*p*<0.05) are denoted by asterisks (*) in (b). Canonical correlation analysis (CCA; [c]) was used to model differences between outliers (right centroid) and non-outliers (left centroid), and all 11 outliers are labeled in (c). There was a statistically significant correlation between the canonical score (axis 1) and the Mahalanobis distance (d), and six clear outliers have been labeled in the scatterplot. The following letters behind the outlier sample identification numbers denote the site of collection (Table 1) in (c)-(d): a = FJTO05, b = FJTO07, c = FJMT13, d = FJML18, e = FJFL29, f = FJKB31, g = FJMG51, h = FJVB52, i = FJVB55, j = FJVB63, and k = FJVB67.

Upon providing a brief treatise of the environmental data (Table 1, Results A in S1 File and S1 Data File) and host genotype frequency results (Fig 2; “Island descriptions, coral cover, and host genotype breakdown”), we then proceed to discuss the analysis of the physiological and molecular data. First, we discuss the results of the univariate ANOVAs and MANOVAs aimed at uncovering the effects of environment on coral molecular physiology (“Univariate statistical analysis and multivariate ANOVA;” Table 2; analytical aim 1). We then talk about the MSA used to visualize variation in the dataset (PCA; Fig 3A and 3B) and similarity between samples (MDS+ANOSIM; Fig 3C). Both methods were able to uncover outliers (analytical aim #2), albeit not in a quantitative manner. In contrast, the Mahalanobis distance (Fig 3D) is a more quantitative means of calculating deviation from a local norm, and this method was used in combination with the heat map score (visually depicted in Fig 4A and explicitly stated in Table 3) to determine which samples were outliers (Table 3). Statistical tests of outlier frequency across environment can be found in Table 2. Student’s *t*-tests were used to detect differences between outliers and non-outliers (“Outliers vs. non-outliers;” Table 3; analytical aim 3), and a predictor screening algorithm was used to rank the response variables in terms of their proportional contribution to the overall difference between these two groups of samples (Fig 4B). CCA (Fig 4C) was used to graphically depict differences between outliers and non-outliers (“Canonical correlation analysis of outliers vs. non-outliers”), and the canonical scores from this analysis were regressed against the respective Mahalanobis distance to portray the congruency in these two test statistics (Fig 4D). Finally, MANCOVA (“Outlier multivariate analysis of covariance”) and ANCOVA (“Outlier analysis of covariance”) were used to model differences in the relationships amongst certain response variables between outliers and non-outliers (Table 4). The details of each of these analyses are described in detail below.

**Table 2:** 1-way ANOVA + multivariate ANOVA (MANOVA) matrix for data pooled across coral species. The values below the environmental parameters (EP; top row) represent the number of categorical groupings. *X*-squared tests and 1-way ANOVAs were used to analyze the frequency (freq.) and molecular-physiological response variable (MPRV) data, respectively. Although the Bonferroni-adjusted α level was 0.004, all *p*-values less than 0.05 have been presented. The multivariate means were analyzed with MANOVA, though the same, Bonferroni-adjusted α level was used. Colony color was excluded from this analysis (though it was considered as a MPRV for the species-specific matrices [S4 Table]); color did vary marginally across islands (1-way ANOVA, *p* = 0.05). temp. = temperature. PAR = photosynthetically active radiation. Sym = *Symbiodinium*. TFS = too few samples to conduct statistical test. NS = not statistically significant.

EP (n = 14)/ response	island (n = 9)	site (n = 35)	exposure (n = 3)	reef zone (n = 3)	reef type (n = 4)	date (n = 21)	time (n = 3)	depth (n = 7)	temp. (n = 2)	salinity (n = 9)	PAR (n = 4)	ALCC (n = 5)	host (n = 5)	Sym assem-blage (n = 3)
host freq.	*p*<0.001	TFS	*p*<0.01	NS	NS	TFS	NS	NS	*p*<0.01	*p*<0.001	NS	*p*<0.001	*—*	NS
outlier freq.	NS	TFS	NS	NS	NS	TFS	NS	NS	NS	NS	NS	NS	NS	NS
**MPRV** (n = 13)														
max. length#	NS	*p*<0.001	NS	NS	NS	NS	NS	NS	NS	NS	NS	NS	*p*<0.004	NS
planar SA#	NS	*p*<0.001	NS	NS	NS	*p*<0.05	NS	NS	NS	NS	NS	NS	*p* = 0.002	NS
Sym GCP	NS	*p*<0.05	*p*<0.01	NS	*p*<0.05	*p*<0.01	NS	NS	NS	NS	NS	NS	NS	NS
RNA/DNA+	NS	NS	NS	NS	NS	NS	NS	NS	NS	NS	NS	NS	NS	NS
Sym *rbcL*#	NS	NS	NS	NS	NS	NS	NS	NS	NS	NS	NS	NS	NS	NS
Sym *zifl1l*+	*p*<0.05	*p*<0.01	NS	NS	NS	*p*<0.05	*p*<0.001	NS	NS	*p*<0.001	NS	NS	NS	NS
Sym *hsp90*+	NS	NS	NS	NS	NS	NS	NS	NS	NS	NS	NS	NS	NS	NS
Sym *ubiq-lig*#	NS	NS	NS	NS	NS	NS	NS	NS	NS	NS	NS	NS	NS	NS
Sym *apx1*#	NS	NS	NS	NS	NS	NS	NS	NS	NS	NS	NS	NS	NS	NS
host *ca*+	NS	NS	NS	NS	NS	NS	NS	NS	NS	NS	NS	NS	NS	NS
host *lectin*#	NS	NS	NS	NS	NS	NS	NS	NS	NS	NS	NS	NS	NS	NS
host *cu-zn-sod*+	NS	NS	NS	NS	NS	*p*<0.05	NS	NS	NS	NS	NS	NS	*p*<0.004	NS
host *gfp-cp*+	*p*<0.01	NS	NS	NS	NS	NS	NS	NS	NS	NS	NS	NS	NS	NS
multivariate mean	*p*<0.01	*p*<0.05	NS	*p*<0.05	NS	*p* = 0.0001	NS	NS	NS	NS	NS	NS	*p*<0.05	NS

+log-transformed data.

#rank-transformed data.

**Table 3:** The eleven outliers identified in the Lau Archipelago dataset. Gene expression data have been presented as non-normalized (2^-Ct^*10^9^) in all but the last two rows; this allows for the back-calculation of the raw threshold cycle (Ct) values so that the typical range of expression of each gene can be more easily assessed by interested individuals. The sample number fraction following the island name represents the number of outliers over the total number of samples for which a Mahalanobis distance could be calculated (rather than the number of samples analyzed from that site). Values representing aberrant levels for a particular response variable (i.e., that contributed to the heat map score) have been highlighted in bold. When there was a statistically significant difference (student’s *t*-test, *p*<0.05) between the outlier and non-outlier averages for a parameter (instead using normalized gene expression data), the lower of the two values has been underlined. No outliers were detected amongst the colonies sampled from Tuvuca (n = 8 samples analyzed in full) and Cicia (n = 8 samples analyzed in full). Fulaga sample 54 was also determined to be an outlier after imputation of missing data (discussed in the main text), though it is not featured in this table. In the “Color” column, the values are as follows: 1 = normal, 2 = pale, 3 = very pale, and 4 = bleached. PAR = photosynthetically active radiation. SA = surface area. GCP = genome copy proportion. Ma Dis = Mahalanobis distance. “.” = missing data.

Island/sample	species	Coll-ection time	Colo-ny depth (m)	PAR (μmolm^-2^s^-1^)	Co-lor	Max. length (cm)	Pla-nar SA (cm^2^)	Sym GCP	RNA/DNA	*Symbiodinium* genes					Host coral genes				MaDis	Heat map score
										*rbcL*	*zifl1l*	*hsp90*	*ubiq-lig*	*apx1*	*ca*	*lec-tin*	*cu-* *zn-* *sod*	*gfp-cp*		
**Totoya** (2/9 samples were outliers; 22%)																				
7	*P*. *damicornis*	11:00	3.7	411	1	21	230	0.27	1.0	181	84.3	31.9	1.58	6.64	68.5	10.1	10.1	**2890**	7.0	1
15	*P*. *damicornis*	9:45	7.0	121	1	7	36	0.23	0.44	42.1	58.2	43.1	0.869	3.73	55.6	**68.5**	8.96	**1950**	4.4	2
**Matuku** (1/9 samples were outliers; 11%)																				
25	*P*. *verrucosa*	8:55	10.0	82	1	7	38	0.17	0.51	**326**	29.8	66.9	1.74	13.6	61.0	40.2	5.85	101	5.5	1
**Moala** (1/10 samples were outliers; 10%)																				
39	*P*. *verrucosa*	10:58	11.3	109	1	59	1600	0.36	1.6	999	22.6	131	6.79	16.7	775	**198**	14.2	88.3	4.4	2
		**ec-tion time**	**ny** **depth** **(m)**	(μmolm^-2^s^-1^)	lor	length (cm)	nar SA (cm^2^)	GCP	DNA	*rbcL*	*zifl1l*	*hsp90*	*ubiq-lig*	*apx1*	*ca*	*lec-tin*	*cu-* *zn-* *sod*	*gfp-cp*		
**Fulaga** (1/6 samples were outliers; 17%)																				
56	*P*. *acuta*	16:06	2.5	.	2	16	120	0.14	0.54	**337**	18.3	**122**	**3.40**	9.39	157	8.36	**61.0**	353	5.2	4
**Kabara** (1/4 samples were outliers; 25%)																				
68	*P*. *acuta*	15:53	14.4	17	2	17	140	0.13	0.45	244	8.36	**88.3**	**2.40**	5.91	21.1	55.6	**65.4**	287	4.4	3
**Mago** (1/7 samples were outliers; 14%)																				
111	*P*. *acuta*	13:36	5.6	.	1	18	170	0.13	0.39	488	49.5	73.4	**4.80**	14.6	116	12.4	82.4	378	4.7	1
**Vanua Balavu** (4/18 samples were outliers; 22%)																				
115	*P*. *damicornis*	10:26	7.0	123	2	11	70	0.13	0.29	238	**706**	99.1	2.40	6.19	1202	22.6	68.5	523	8.2	1
123	*P*. *acuta*	10:03	8.7	174	1	19	170	0.44	0.29	287	56.9	194	4.13	20.6	**1170**	53.7	**125**	222	5.8	2
146	*P*. *acuta*	9:28	9.6	140	1	17	170	0.04	0.18	62.4	8.36	**75.1**	**0.830**	4.92	38.4	2.19	16.7	40.2	8.0	2
153	*P*. *acuta*	14:50	21.1	49	1	12	71	0.11	**1.7**	**256**	7.99	37.5	1.62	6.19	**1350**	12.4	50.7	511	5.5	3
**Outlier avg. (normalized data**±std. dev.**)**			9.2 ± 5.2	137 ± 114	1.3± 0.47	19 ± 15	252 ± 437	0.19 ± 0.12	0.67 ± 0.53	6x10^4^± 3x10^4^	2x10^4^± 3x10^4^	2x10^4^± 2x10^4^	540 ± 290	61 ± 33	2x10^4^± 2x10^4^	2x10^3^± 2x10^3^	1700± 1300	4x10^4^± 6x10^4^	5.7± 1.4	2 ± 1
**Non-outlier avg. (normalized data**±std. dev.**)**			11.3 ± 6.4	123 ± 120	1.5± 0.87	17 ± 11	187 ± 289	0.29 ± 0.13	0.76 ± 0.43	2x10^4^± 2x10^4^	4x10^3^± 6x10^3^	5x10^3^± 5x10^3^	170 ± 110	36 ± 29	10^4^ ± 9x10^3^	10^3^ ± 10^3^	620 ± 504	10^4^ ± 2x10^4^	2.5± 0.69	0.2 ± 0.1

**Table 4:** Analysis of covariance. Since MANOVA revealed that a negative relationship between biological composition (the RNA/DNA ratio and the *Symbiodinium* genome copy proportion [GCP]) and gene expression significantly distinguished outliers from non-outliers (Fig 4C), multivariate analysis of covariance (MANCOVA) and univariate ANCOVA were performed on the multivariate means (excluding size data) and individual gene expression means, respectively. Only genes for which statistically significant interaction effects were documented between a biological composition parameter and outlier status (analyzed as a categorical variable: outlier [yes] vs. non-outlier [no]) have been presented. For MANCOVA, Wilks’ lambda values are shown for comparisons between a continuous variable and a categorical one, while Exact *F* values are shown between two continuous variables. For the multivariate data, individual correlations were tested between canonical scores (first axis only) and 1) the *Symbiodinium* GCP and 2) the RNA/DNA ratio. *t* = linear regression test statistic. *p<0.05. **p<0.01. ***p<0.0001.

Parameter Test	Wilks’ lambda/ Exact *F*	Out-lier *r*^2^	Outlier *t*	Non-outlier *r*^*2*^	Non-outlier *t*	Finding(s)
**Multivariate mean** (MANCOVA)	0.065***					
Outlier (yes vs. no)	0.382***					See Fig 4C.
*Symbiodinium* GCP	9.30***	0.72	-4.80***	0.14	-3.05**	Strong negative relationship between gene expression and *Symbiodinium* GCP (density) for outliers, though only a weak relationship for non-outliers.
Outlier x *Symbiodinium* GCP	0.393***					
**Multivariate mean** (MANCOVA)	0.152***					
Outlier (yes vs. no)	11.8***					
RNA/DNA	6.56***	0.04	-0.57	0.09	2.36*	Weak positive relationship between gene expression and RNA/DNA ratio for non-outliers, but not for outliers.
Outlier x RNA/DNA	3.12**					
***Symbiodinium rbcL* mRNA expression** (ANCOVA)						
Outlier (yes vs. no)	7.33**					See Table 3.
*Symbiodinium* GCP	13.5***					
RNA/DNA	4.29*					
*Symbiodinium* GCP x outlier	4.92**	0.34	-2.15	0.03	-1.28	More strongly negative relationship between *rbcL* mRNA expression and *Symbiodinium* GCP (density) for outliers than for non-outliers.
RNA/DNA x outlier	0.682	0.02	0.41	0.05	1.78	
***Symbiodinium ubiq-lig* mRNA expression** (ANCOVA)						
Outlier (yes vs. no)	10.7***					See Table 3.
*Symbiodinium* GCP	24.5***					
RNA/DNA	3.20					
*Symbiodinium* GCP x outlier	8.60**	0.49	-2.97*	0.04	-1.58	More strongly negative relationship between *ubiq-lig* mRNA expression and *Symbiodinium* GCP (density) for outliers than for non-outliers.
RNA/DNA x outlier	1.80	0.04	-0.58	0.02	1.04	
***Symbiodinium hsp90* mRNA expression** (ANCOVA)						
Outlier (yes vs. no)	6.03*					See Table 3.
*Symbiodinium* GCP	20.9***					More strongly negative relationship between *hsp90* mRNA expression and *Symbiodinium* GCP (density) for outliers than for non-outliers.
RNA/DNA	4.98*					
*Symbiodinium* GCP x outlier	18.0***	0.37	-2.31*	0.01	-0.75	More strongly negative relationship between *hsp90* mRNA expression and RNA/DNA for outliers than for non-outliers.
RNA/DNA x outlier	5.31*	0.17	-1.34	<0.00	0.07	

### Island descriptions, coral cover, and host genotype breakdown

As this represents the first comprehensive survey of coral reefs of Fiji’s Lau Archipelago, detailed site descriptions have been provided for each island/atoll in the Results A in S1 File. ALCC and other environmental data can be found in Table 1, though for a more detailed treatise of the coral cover data, please see the Results A in S1 File. To peruse all environmental data, and not just those of the sites from which corals were collected, please see the S1 Data File. To see coral reef habitat images for each site, please click on the “Fiji” sub-heading of the following website: coralreefdiagnostics.com. ALCC of Lau’s reefs was 33±13% (std. dev.), and it varied significantly across islands (1-way ANOVA, *p*<0.0001). Of the corals sampled and genotyped, host assemblage (i.e., freq.) also differed significantly across islands (Table 2), and ~ 1/3 of the sampled colonies were *not* the model coral *P*. *damicornis*, but instead its closely related sister species *P*. *acuta* (Fig 1).

### Univariate statistical analyses and multivariate ANOVA

At the Bonferroni-adjusted α of 0.004, few environmental factors affected coral physiology (Table 2). Max. colony length and planar SA differed significantly across species, due in part to some abnormally large *P*. *verrucosa* colonies (S1 and S3 Tables); indeed, *P*. *verrucosa* was, on average, bigger (greater max. length and planar SA) than all other species (Tukey’s honestly significant difference [HSD], *p*<0.05) except *P*. *brevicornis*. Host coral *cu-zn-sod* mRNA expression also differed significantly across the five host species (Table 2), with *P*. *acuta* expressing significantly higher levels than *P*. *damicornis* (~2-fold difference) and *P*. *verrucosa* (3-fold difference; Tukey’s HSD, *p*<0.05 for both comparisons). *Symbiodinium zifl1l* mRNA expression varied significantly over time (Table 2); expression was ~9-fold higher at the <10:00 and 10:00–14:00 intervals than after 14:00 (Tukey’s HSD, *p*<0.05 for both comparisons).

At the Bonferroni-adjusted α level of 0.002 (please note that this adjustment differs from that when data were pooled across species.), there were also few statistically significant effects of environment on the 13 response variables for any of the three most commonly sampled species analyzed individually- *P*. *damicornis*, *P*. *acuta*, and *P*. *verrucosa* (S4 Table). First, *zifl1l* mRNA expression in *Symbiodinium* populations of *P*. *verrucosa* varied significantly across sampling times, with lowest expression levels measured in colonies sampled after 14:00 (S4 Table). The mean max. length of colonies sampled between 5 and 10 m was less than that of those sampled <5 m and between 10 and 15 m (Tukey’s HSD, *p*<0.05 for both comparisons; S4 Table). Finally, MANOVA of the effect of environment on coral physiology did not unveil any statistically significant differences for data pooled across species (Table 2; the exception being sampling date [see the Results A in S1 File for details.]) or analyzed individually for each of the three aforementioned species (see the Results A in S1 File and S4 Table for details.).

### Principal components analysis

When looking at all 13 molecular physiological response variables (Fig 3A), the first two PC encompassed less than 45% of the variation; PC1 was dominated by *Symbiodinium* mRNAs, while PC2 featured the two size-related parameters as the dominant, positive loading factors (S1 Data File). Although the low percentage of the variation encompassed suggests that PCA may not be the ideal means of identifying response variables that best partitioned the samples, there was nevertheless some separation of a number of *P*. *acuta* samples along PC1, and certain *P*. *verrucosa* samples from Moala were separated along PC2. The most divergent sample evident, Moala 39, was considered such due to its immense size (S1 Table) and not because of aberrant molecular physiology (S3 Table). A second PCA was performed with the 11 molecular-scale response variables only (Fig 3B), and three *P*. *acuta* samples were well separated from the core region of the dataset along PC1: Fulaga 54+56 and Vanua Balavu 146. PC1 was dominated by *Symbiodinium* genes, particularly *ubiq-lig* and *rbcL* (S1 Data File), while two host coral mRNAs (*lectin* and *cu-zn-sod*) had the highest positive loading scores in PC2. However, as when all 13 response variables were considered, the first two PC encompassed only 45% of the variation in the dataset.

### Multidimensional scaling and analysis of similarity

A Bray-Curtis similarity matrix was used to construct an MDS plot of the 70 samples for which no data were missing (Fig 3C), and ANOSIM was used to test the effects of the 14 environmental parameters on the resulting dataspace; only 2 such parameters demonstrated a significant influence (*p*<0.05): reef zone and host; however, the stress was over 0.2, so these results should be interpreted cautiously. Furthermore, because 62 of the 70 analyzed samples were from fore reefs, the sample size for the remaining reef zones (lagoon and back reef) was too small to interpret the former difference with confidence. In contrast, there were more than five specimens for four of the five host species (Fig 1; excluding *P*. *brevicornis*), and the *P*. *meandrina* samples appeared to cluster together in the MDS plot (Fig 3C). In contrast, the *P*. *damicornis*, *P*. *acuta*, and *P*. *verrucosa* samples appear intermixed. Finally, one outlier identified by other methods discussed below, Totoya 7, appears well separated from the core region of the MDS plot.

### Outlier frequency vs. environment

When a sample’s 1) Mahalanobis distance (Fig 3D) was above the UCL of 4.29 and 2) heat map score was ≥1 (Fig 4A), it was considered an outlier, and 11 outliers were documented across the 70-sample subset for which no data were missing (S1 Table, S3 Table, and Table 3). A detailed description of the response variables that contributed most significantly to a sample being deemed an outlier can be found in the Results A in S1 File (under “site descriptions”) and S1 and S3 Tables; no environmental parameter significantly affected outlier frequency (Table 2).

### Outliers vs. non-outliers

To understand the overall differences between the 11 outliers and the 59 non-outliers, a series of student’s *t*-tests were first performed; several notable differences were unveiled (Table 3). First, although outliers and non-outliers were of similar size (however, size was not included in the outlier assignment exercise.), the former had ~30% lower *Symbiodinium* densities (Table 3). Furthermore, outliers demonstrated higher expression levels of six of the nine target genes, including all but one (*apx1*) of the *Symbiodinium* genes. In general, then, outliers had 30% lower *Symbiodinium* densities and 3-4-fold higher expression levels of the following six genes: *Symbiodinium rbcL*, *hsp90*, *ubiq-lig*, and *zifl1l* and host coral *cu-zn-sod* and *gfp-cp* (Table 3).

### Predictor screening

As another means of uncovering the response variables that best differentiated outliers from non-outliers, JMP’s predictor screening function was used to rank the response variables in terms of their proportional contribution to the cumulative difference between outliers and non-outliers (Fig 4B). *Symbiodinium ubiq-lig* mRNA expression ranked highest, accounting for over 20% of the cumulative difference between outliers and non-outliers. This is unsurprising given the statistically significant, 3-fold difference between outlier *ubiq-lig* expression and non-outlier expression of this gene (Table 3). Similarly, the parameters for which there was no statistically significant difference in the outlier vs. non-outlier student’s *t*-tests of Table 3 ranked lowest: host *ca* (1% of the cumulative difference), RNA/DNA ratio (3%), and host *lectin* (3.5%). In general, *Symbiodinium* genes contributed more to the cumulative difference between outliers and non-outliers than did host coral genes, a trend that was also documented by outlier CCA and MANCOVA (both of which are described below).

### Canonical correlation analysis of outliers vs. non-outliers

CCA featuring 11 of the 13 response variables (excluding the two size parameters) was also used to determine which parameters (or combinations thereof) best separated outliers from non-outliers (Fig 4C), and there was good separation along CA1. In general, the target genes were positively weighted while the biological composition parameters (the RNA/DNA ratio and *Symbiodinium* GCP) were negatively weighted (S1 Data File); in other words, a negative relationship between gene expression and biological composition best separated outliers from non-outliers. Indeed, *Symbiodinium* density was the only factor that was significantly higher in non-outliers (Table 3); all other response variables for which significant differences were uncovered were documented at higher levels in the outliers. Furthermore, *Symbiodinium* density was significantly and negatively correlated with expression of the following *Symbiodinium* genes: *rbcL* (*r*^2^ = 0.16; *p*<0.001), *ubiq-lig* (*r*^2^ = 0.19; *p*<0.001), *apx1* (*r*^2^ = 0.11; *p*<0.01), and *hsp90* (*r*^2^ = 0.11; *p*<0.01). In contrast, it was significantly and positively correlated with expression of the following two host coral genes, albeit weakly: *ca* (*r*^2^ = 0.07; *p* = 0.01) and *lectin* (*r*^2^ = 0.07; *p* = 0.01).

### Outlier multivariate analysis of covariance

To gain more insight into the observation that a negative relationship between biological composition and gene expression best distinguished the 11 outliers from all other samples, MANCOVA was performed (Table 4); it revealed a statistically significant interaction between the *Symbiodinium* density (analyzed as a continuous variable) and outlier status (analyzed as a categorical variable; outlier vs. non-outlier) on the multivariate mean. A statistically significant interaction effect of RNA/DNA x outlier status was also revealed by MANCOVA (Table 4).

### Outlier analysis of covariance

When using ANCOVA to highlight individual genes that drove the MANCOVA response (Table 4), the slopes of the best-fit lines between *Symbiodinium* density and gene expression were significantly different between outliers and non-outliers for the following three *Symbiodinium* genes: *rbcL*, *ubiq-lig*, and *hsp90*. For the latter two genes, there was a statistically significant, negative correlation between mRNA expression and *Symbiodinium* GCP for the outliers, while no such trend existed for the non-outliers. For *rbcL*, the slopes between mRNA expression and *Symbiodinium* density were not statistically significant for either outliers or non-outliers, though the slopes did differ significantly from each other. As final evidence that CCA was able to separate outliers from non-outliers in a statistically meaningful manner, the canonical scores were regressed against the Mahalanobis distance for all samples (Fig 4D), and a statistically significant, positive correlation was obtained; samples with large Mahalanobis distances (i.e., outliers) were more likely to also have high canonical scores along CA1.

## Discussion

### The relationship between *Symbiodinium* density and gene expression

A combination of univariate and multivariate statistical approaches were used herein to uncover corals displaying statistically aberrant behavior. Interestingly, two exploratory approaches used to depict variation in the dataset and similarity amongst samples (PCA and MDS, respectively) were able to uncover several colonies positioned away from the normal “core” physiological response region; all such samples were ultimately found to be outliers based on a more quantitative approach featuring the Mahalanobis distance and the heat map score. These 11 outliers had ~30% lower *Symbiodinium* densities and 3- to 4-fold higher stress gene expression levels than non-outliers, and the negative relationship between *Symbiodinium* density and expression of both *hsp90* and *ubiq-lig* was much more pronounced for outliers than non-outliers. Reef-building corals require high densities (~10^6^ cells cm^-2^) of *Symbiodinium* to maintain their metabolic needs [36]. As environments change, particularly with respect to temperature, low levels of bleaching can take place [37], resulting in lower densities of *Symbiodinium in hospite*. Such a hypothetical, bleaching-inducing environmental change would likely also affect cell physiology, specifically the expression of genes encoding stress proteins (such as *hsp90* and *ubiq-lig*). Therefore, it is unsurprising that stress-sensitive genes were expressed at higher levels in corals exhibiting lower *Symbiodinium* densities.

### Outlier frequency and environment

In contrast to what was hypothesized, there was no effect of any environmental parameter on outlier frequency; corals displaying statistically aberrant behavior were just as likely, for instance, to be found in the lagoon as on the fore reef. In fact, there were numerous instances in which outliers were sampled from a reef in which normally behaving corals were also sampled. For instance, of the six corals sampled at site FJMT13 (Matuku), only one was considered an outlier (sample 25). Another member of the same species (*P*. *verrucosa*) of nearly identical size was collected within ~100 m at nearly the same depth and light level, and this sample was deemed normal with respect to the 11 molecular physiological response variables. Therefore, it is possible that intra-site environmental variation, or, alternatively, differing life histories, led to the aberrant behavior of colony 25, which appeared normally pigmented (albeit only 7-cm long and therefore presumably young).

There was only one site in which multiple outliers were sampled: FJFL29 (a lagoonal patch reef at Fulaga); although only one such outlier is listed for Fulaga (colony 56) in Table 3, when looking at the heat map scores, it is clear that colony 54 was likely an outlier. It could not be labeled as such because of to the inability to calculate its respective Mahalanobis distance due to poor DNA extraction efficiency. Therefore, JMP’s “multivariate normal” imputation algorithm featuring a shrinkage estimate was used to impute missing data (with off-diagonals scaled by a factor of 0.75), and the corresponding Mahalanobis distance for sample 54 was 8.6, the highest value in the entire dataset. Samples 54 and 56 were collected within 10 minutes of each other (~16:00) in <3 m of water in an area characterized by such high sediment loads (not quantified) that visibility was <1 m; such sedimentation may have contributed to the aberrant sub-cellular behavior of these samples either directly (e.g., by smothering the tissues and therefore necessitating a stress response [thereby affecting *ubiq-lig*]) or indirectly (e.g., via modification of the corals’ light environment [thereby affecting *rbcL*]).

### Biomarker profiling in the absence of pristine control reefs

Comparisons with biomarker expression signatures of samples from controlled tank studies conducted elsewhere (e.g., [38–41]) are risky, as what is considered a control level of expression for a certain target molecule in a region like Taiwan, whose reefs abut some of the world’s highest human population densities, may actually be “stress-indicative” in a place like Lau Province; although the reefs of the Lau Archipelago are far from pristine due in part to a virtual absence of sea cucumbers from over-harvesting by Chinese fleets (unpublished data), the region is only sparsely populated (~11,000 people across the 60 islands, only about half of which are populated). However, as documented in Southern Taiwan [15] and even uninhabited South Pacific atolls (e.g., Maria Atoll, French Polynesia; [14]), all 70 of the Lau samples expressed high levels of stress marker genes, including the *Symbiodinium* stress genes *ubiq-lig* and *hsp90* (but not *apx1*) and the host coral stress genes *cu-zn-sod* and *gfp-cp*. Although the latter is not a classical stress gene, per se, in corals it is known to be up-regulated at high PAR levels [42]; the respective chromoproteins absorb excess light that might otherwise lead to photoinhibition [43] and consequently bleaching. Whether or not these generally high levels of expression of genes encoding stress proteins indicates that these corals were indeed stressed at the time of sampling or were, alternatively, better prepared for future environmental changes (as discussed in the Introduction), remains to be determined. Furthermore, little congruency between mRNA and protein expression was documented for another reef-building pocilloporid [40]; therefore, it is possible that the respective proteins may show entirely different expression patterns.

If nine of ten corals on a reef display very similar molecular phenotypes, whereas the latter (i.e., the “outlier”) is characterized by a completely different one, this does not necessarily mean that the outlier is stressed and the former nine colonies are healthy; such a guess could only be made if there existed a detailed knowledge of the environmental history of the samples, or, alternatively, if the corals’ growth and reproductive output were monitored over a multi-week timescale. Therefore, it is not currently possible to state whether the 11 outliers identified herein were stressed, despite their being characterized by lower *Symbiodinium* densities and higher stress gene expression; it can only be stated with the data in hand that they were behaving significantly differently from the other 59 colonies analyzed. It would be fruitful to return to the same sites at which both outliers and non-outliers were found, such as FJFL29, during a period of anomalously high temperatures to see if the outliers are actually of diminished resilience to environmental change than the conspecific non-outliers. If such were found to be the case, the validity of this approach for use as a coral stress test would be substantiated.

### Future directions in coral health assessment

Although at current average costs (~US$150/sample, excluding bioinformatics costs), next generation sequencing technology was prohibitively expensive for analyzing these 70 samples (in comparison to ~US$30/sample spent to assess the 11 molecular-scale response variables herein), sequencing prices will continue to drop, and so it may ultimately be possible to profile entire transcriptomes of several dozen, or even hundred, coral samples in the coming years at reasonable costs. Then, it may be found that there are mRNAs that are *only* expressed by truly stressed colonies (as determined by tank experiments in which growth, reproductive output, and *Symbiodinium* densities/pigmentation are also tracked over a long-term timescale); if such a result could be corroborated in conspecifics sampled across numerous study sites around the globe, then it is conceivable that a molecular biomarker assay for conclusive determination of coral health could ultimately be developed. In that case, the conceptual and statistical framework reported herein could be used in conjunction with such biomarkers in order to not only label a coral as being an outlier or not, but, more specifically, to assign each coral sample of interest a health index score (e.g., from 1 to 10, with 1 being nearly dead and 10 being healthy).

## Supporting information

S1 Data File Spreadsheet featuring all data presented in the manuscript. Separate worksheets are presented for 1) environmental data only (“site data”), 2) environmental, physiological, and molecular data (“all data”), and 3) principal components analysis (PCA) and canonical correlation analysis (CCA) results (“PCA+CCA”). Please note that the physiological and molecular data from the sample corals have been presented as *Z*-scores; raw data can be provided upon request.
(XLSX)

S1 Table Sample information I-environmental, size, and biological composition data. Site information and other environmental data can be found in Table 1. The fraction behind the island name represents the number of samples processed for molecular physiological response variables over the total number of colonies sampled. All samples hosted *Symbiodinium* of clade C only unless otherwise noted. The size data (maximum [max.]. length and planar surface area [SA]) were not considered in the calculation of the Mahalanobis distance, though they *were* considered in the calculation of the multivariate means (Table 2). There were significant effects of island for parameters underlined in bold (see Table 1.), though *post-hoc* differences were revealed for “Collection PAR,” max. length, and planar SA only, in which case Tukey’s honestly significant difference groups (*p*<0.05; as lower-case letters) have been placed behind the standard deviation for each island. Average values from the Austral and Cook Islands dataset [2][11] were underlined when they differed significantly from those of Fiji (student’s *t*-test, effect of region, *p*<0.05). For outliers (highlighted in blue), the value(s) for the biological composition parameter(s) that had *Z*-scores <-2 or >2 has/have been highlighted in bold font; when neither biological composition parameter is highlighted for an outlier, this means that gene expression data (S3 Table) instead contributed to the high Mahalanobis distance value and heat map score. Color was scaled as normal = 1, pale = 2, very pale = 3, or bleached = 4. PAR = photosynthetically active radiation (μmol photons m^-2^ s^-1^). mORF = mitochondrial open reading frame. GCP = genome copy proportion. “.” = missing data. MD = could not be calculated due to “missing data.”
(DOCX)

S2 Table Target genes and real-time PCR conditions. All assays utilized SYBR® Green chemistry except for the Solaris™ RNA spike, which required the use of a proprietary Taqman® probe provided by the manufacturer (described in the Methods A in S1 File). Corals characterized by either highly elevated or severely diminished expression levels of the target genes were hypothesized to be displaying aberrant behavior at the time of sampling (see the main text for details.). bp = base pairs. *p<0.05. **p<0.001.
(DOCX)

S3 Table Sample information II-gene expression data. To calculate the principal component (PC) score (first axis only; PC1), principal components analysis (PCA) was performed on the 11 molecular-scale response variables only (i.e., excluding size data, but including biological composition data [RNA/DNA ratio and *Symbiodinium* genome copy proportion (GCP)]). The global mean *r*^2^ between the PC1 score and the Mahalanobis distance was 0.40, and this positive correlation was statistically significant (linear regression *t*-test, *p*<0.001). When a sample was considered an outlier, the value(s) for the response variable(s) that had *Z*-scores <-2 or >2 has/have been highlighted in bold font. Frequencies (freq.) in the “Outlier?” column represent the number of outliers over the total number of samples for the respective island with enough data to calculate the Mahalanobis distance; please note that this may be lower than the total number of samples analyzed for that island. When gene expression varied significantly across islands within the Lau Archipelago (see Table 2 for ANOVAs conducted with normalized data.), the gene name has been highlighted in bold font, and for *cu-zn-sod*, Tukey’s honestly significant differences were detected (*p*<0.05; denoted by lower-case letters). When a significant difference (student’s *t*-test, *p*<0.05) was detected between the two regions (Lau Province, Fiji vs. Austral Islands, French Polynesia+Cook Islands [11]), the lower of the two means has been underlined. The “Maximum/minimum” fold difference value in the final row was calculated by dividing the highest expression level of the dataset by the lowest. Ct = threshold cycle. “.” = missing data. MD = value could not be calculated due to “missing data.” NA = not assessed.
(DOCX)

S4 Table Univariate and multivariate ANOVAs (MANOVAs) of the *Pocillopora acuta*, *P*. *damicornis*, and *P*. *verrucosa* datasets. Site and date were excluded from the analysis due to typically having too few samples (TFS) for a robust comparison. Unlike in the univariate comparisons pooled across species (Table 2), color was included in this species-specific analysis (n = 12 environmental parameters). Photosynthetically active radiation (n = 4 categorical groupings) was included in the analysis but excluded from the table, as it did not significantly affected any response variable for any of the three species. Likewise, only molecular physiological response variables (MPRV) for which at least one environmental parameter (EP) led to a significant difference (non-Bonferroni-adjusted) have been included in the table; as an exception, the multivariate centroid results have been included even when all were negative for a particular species (e.g., *P*. *damicornis* and *P*. *verrucosa*). Since 13 response variables were assessed across three species and 12 environmental parameters, 468 ANOVAs were performed; therefore a Bonferroni adjustment of 22 was made to the α level of 0.05, resulting in a multiple comparisons-adjusted α of 0.002; few results were statistically significant at this level, and those that were have been highlighted in green. The island number is listed as “variable” since not all species were found at each island. NS = not significant. NA = not applicable. ALCC = average live coral cover.
(DOCX)

S1 File Supplemental methods and results. (DOCX)
