# Supplementary material for: Identifying corals displaying aberrant behavior in Fiji’s Lau Archipelago
Source: PLoS One. 2017 May 24;12(5):e0177267. doi: 10.1371/journal.pone.0177267 (PMC5443480; doi:10.1371/journal.pone.0177267)
Supplement: S1 Table — Site information and other environmental data can be found in Table 1. The fraction behind the island name represents the number of samples processed for molecular physiological response variables over the total number of colonies sampled. All samples hosted Symbiodinium of clade C only unless otherwise noted. The size data (maximum [max.]. length and planar surface area [SA]) were not considered in the calculation of the Mahalanobis distance, though they were considered in the calculation of the multivariate means (Table 2). There were significant effects of island for parameters underlined in bold (see Table 1.), though post-hoc differences were revealed for “Collection PAR,” max. length, and planar SA only, in which case Tukey’s honestly significant difference groups (p<0.05; as lower-case letters) have been placed behind the standard deviation for each island. Average values from the Austral and Cook Islands dataset [2][11] were underlined when they differed significantly from those of Fiji (student’s t-test, effect of region, p<0.05). For outliers (highlighted in blue), the value(s) for the biological composition parameter(s) that had Z-scores <-2 or >2 has/have been highlighted in bold font; when neither biological composition parameter is highlighted for an outlier, this means that gene expression data (S3 Table) instead contributed to the high Mahalanobis distance value and heat map score. Color was scaled as normal = 1, pale = 2, very pale = 3, or bleached = 4. PAR = photosynthetically active radiation (μmol photons m-2 s-1). mORF = mitochondrial open reading frame. GCP = genome copy proportion. “.” = missing data. MD = could not be calculated due to “missing data.” (DOCX) [file pone.0177267.s002.docx]

**S1 table. Sample information I-environmental, size, and biological composition data.** Site information and other environmental data can be found in Table 1. The fraction behind the island name represents the number of samples processed for molecular physiological response variables over the total number of colonies sampled. All samples hosted *Symbiodinium* of clade C only unless otherwise noted. The size data (maximum [max.]. length and planar surface area [SA]) were not considered in the calculation of the Mahalanobis distance, though they *were* considered in the calculation of the multivariate means (Table 2). There were significant effects of island for parameters underlined in bold (see Table 1.), though *post-hoc* differences were revealed for photosynthetically active radiation (“Collection PAR;” μmol m^-2^ s^-1^), max. length, and planar SA only, in which case Tukey’s honestly significant difference groups (*p*<0.05; as lower-case letters) have been placed behind the standard deviation for each island. Average values from the Austral and Cook Islands dataset [2][11] were underlined when they differed significantly from those of Fiji (student’s *t*-test, effect of region, *p*<0.05). For outliers (highlighted in blue), the value(s) for the biological composition parameter(s) that had *Z*-scores <-2 or >2 has/have been highlighted in bold font; when neither biological composition parameter is highlighted for an outlier, this means that gene expression data (S3 table) instead contributed to the high Mahalanobis distance value and heat map score. Color was scaled as normal=1, pale=2, very pale=3, or bleached=4. mORF=mitochondrial open reading frame. GCP=genome copy proportion. “.” = missing data. MD=could not be calculated due to “missing data.”

| **Island** | Host | Collection  time | **Colony depth** | **Collection PAR** | Color | NCBI accession for mORF | **Max. length** | **Planar**  **SA** | **Sym GCP (unit-less)** | RNA/ DNA (unit-less) | Mahala-nobis distance | Heat map score |
| --- | --- | --- | --- | --- | --- | --- | --- | --- | --- | --- | --- | --- |
| Sample | |  | **(m)** |  |  | sequence | **(cm)** | **(cm^2^)** |  |  |  |  |
| **Totoya** (13 analyzed/20 collected) | | | | | |  |  |  |  |  |  |  |
| 2 | . | 10:35 | 6.6 | 217 | 1 | . | 13 | 79 | + | . | MD | MD |
| 3 | *P. damicornis* | 10:50 | 5.0 | 312 | 1 | KP942653 | 11 | 58 | 0.35 | 0.23 | MD | 0 |
| 4 | *P. damicornis* | 15:10 | 10.6 | 72 | 1 | KR919830 | 21 | 204 | 0.25 | 0.56 | 3.7 | 0 |
| 5 | *P. damicornis* | 15:25 | . | 117 | . | KR919827 | 14 | 140 | 0.18 | . | 2.4 | 0 |
| 6 | *P. damicornis* | 17:25 | 4.5 | 8 | 3 | KR919823 | 8 | 30 | 0.16 | 0.73 | 2.0 | 0 |
| 7 | *P. damicornis* | 11:00 | 3.7 | 411 | 1 | KR919822 | 21 | 230 | 0.27 | 1.0 | **7.0** | 1 |
| 9 | . | 15:00 | 12.5 | 42 | 1 | . | 14 | 120 | + | . | MD | MD |
| 10 | *P. damicornis* | 15:15 | 10.5 | 168 | 1 | KP942654 | 6 | 25 | 0.41 | 0.78 | 4.2 | 1 |
| 15 | *P. damicornis* | 9:45 | 7.0 | 121 | 1 | KR919825 | 7 | 36 | 0.23 | 0.44 | **4.4** | 2 |
| 16 | *P. meandrina* | 11:10 | 10.0 | 331 | 1 | KR919843 | 8 | 29 | 0.36 | 0.91 | 1.9 | 0 |
| 17 | *P. meandrina* | 11:20 | 11.0 | 364 | 1 | KP942655 | 11 | 60 | 0.31 | 0.72 | 1.5 | 0 |
| 19 | *P. meandrina* | 11:43 | 8.0 | 327 | * | KR919854 | . | . | 0.24 | 0.32 | MD | MD |
| 20 | *P. damicornis* | 11:50 | 7.5 | 460 | * | KR919831 | . | . | 0.51 | 1.0 | 2.2 | 0 |
|  | **Totoya avg.±std. dev.** | | 8.1±2.8 | 227±149^a^ | 1.2±0.63 |  | 12±5.2^b^ | 92±72^b^ | 0.30±0.10 | 0.67±0.27 | 3.3±1.8 | 0.4±0.7 |
| **Matuku** (10 analyzed/10 collected) | | | | | |  |  |  |  |  |  |  |
| 21 | *P. verrucosa* | 10:38 | 11.5 | 144 | 1 | KP942644 | 19 | 230 | 0.20 | 0.63 | 2.5 | 0 |
| 22 | *P. verrucosa* | 10:50 | 11.3 | 127 | 1 | KP942645 | % | % | **0.62** | 0.73 | 2.7 | 1 |
| 23 | *P. verrucosa* | 10:54 | 8.0 | 195 | 1 | KR919833 | 12 | 88 | 0.24 | 0.60 | 2.5 | 0 |
| 24 | *P. damicornis* | 8:50 | 9.0 | 83 | 1 | KR919828 | 15 | 120 | 0.39 | 0.73 | 2.3 | 0 |
| 25 | *P. verrucosa* | 8:55 | 10.0 | 82 | 1 | KR919832 | 7 | 38 | 0.17 | 0.51 | **5.5** | 1 |
| 26 | *P. meandrina* | 9:05 | 7.5 | 90 | 3 | KR092026 | 18 | 160 | 0.28 | 0.40 | 2.6 | 0 |
| 27 | *P. damicornis* | 9:12 | 8.5 | 59 | 1 | KR919824 | 8 | 37 | 0.20 | 1.3 | 3.9 | 1 |
| 28 | *P. damicornis* | 9:22 | 11.0 | 74 | 1 | KR919850 | 16 | 120 | 0.43 | 0.57 | 1.7 | 0 |
| 29 | *P. verrucosa* | 9:26 | 9.5 | 94 | 2 | KP942646 | 7 | 22 | 0.52 | 0.92 | MD | MD |
| 30 | *P. damicornis* | 11:05 | 10.8 | 86 | 4 | KR919845 | 12 | 70 | 0.31 | 0.58 | 2.1 | 0 |
|  | **Matuku** **avg.±std. dev.** | | 9.7±1.4 | 103±41^ab^ | 1.6±1.1 |  | 13±4.6^b^ | 98±67^b^ | 0.34±0.15 | 0.70±0.25 | 2.9±1.2 | 0.3±0.5 |
| **Moala** (12 analyzed/17 collected) | | | | | |  |  |  |  |  |  |  |
| 33 | *P. verrucosa* | 15:47 | 4.0 | 59 | 1 | KP942647 | 16 | 130 | 0.42 | 0.69 | 1.6 | 0 |
| 34 | *P. meandrina* | 15:53 | 4.4 | 31 | 1 | KR919856 | 10 | 47 | 0.27 | 0.25 | 2.2 | 0 |
| 35 | . | 16:00 | 5.0 | 35 | 1 | . | 9 | 33 | + | . | MD | MD |
| 37 | *P. verrucosa* | 10:40 | 11.8 | 136 | 1 | KR919839 | 19 | 130 | 0.38 | 1.6 | 3.0 | 0 |
| 38 | . | 10:50 | 11.1 | . | 1 | . | 14 | 93 | + | . | MD | MD |
| 39 | *P. verrucosa* | 10:58 | 11.3 | 109 | 1 | KP942648 | 59 | 1600 | 0.36 | **2.0** | **4.4** | 2 |
| 40 | *P. verrucosa* | 11:03 | 10.8 | . | 1 | KR919840 | 58 | 1400 | 0.28 | 0.64 | 2.1 | 0 |
| 41 | *P. verrucosa* | 11:14 | 10.3 | 277 | 1 | KR919837 | 56 | 1500 | 0.35 | 0.09 | 2.5 | 0 |
| 42 | *P. verrucosa* | 14:25 | 5.6 | 39 | 1 | KP942649 | 12 | 89 | 0.19 | 0.26 | 1.9 | 0 |
| 44 | *P. acuta* | 16:30 | 6.6 | 39 | 1 | KP942650 | 15 | 140 | 0.49 | 0.28 | 2.5 | 0 |
| 45 | *P. verrucosa* | 8:46 | 15.4 | . | 1 | KP942651 | 41 | 640 | 0.38 | **1.7** | 2.9 | 1 |
| 47 | *P. verrucosa* | 8:55 | 14.2 | . | 1 | KP942652 | 39 | 530 | 0.37 | 1.2 | 1.8 | 0 |
|  | **Moala avg.±std. dev.** | | 9.2±3.9 | 91±85^ab^ | 1.0±0.0 |  | 29±20^a^ | 530±620^a^ | 0.35±0.08 | 0.83±0.64 | 2.5±0.8 | 0.3±0.7 |
| **Fulaga** (7 analyzed/9 collected) | | | | | |  |  |  |  |  |  |  |
| 48 | *P. damicornis* | 9:43 | 4.2 | . | 1 | KP942636 | 14 | 87 | 0.08 | 0.84 | 3.7 | 0 |
| 49 | *P. acuta* | 11:17 | 23 | . | 1 | KP942637 | * | * | 0.31 | 0.92 | 2.9 | 0 |
| 50 | *P. damicornis* | 11:30 | 9.4 | . | 1 | KR919857 | 11 | 63 | 0.27 | 0.19 | 2.1 | 0 |
| 51 | *P. damicornis* | 11:52 | 9.4 | . | 2 | KR919826 | * | * | 0.12 | 0.75 | 2.2 | 0 |
| 54 | *P. acuta* | 15:48 | 2.4 | . | 1 | KR092027 | 27 | 400 | 0.04 | . | MD^$^ | 3 |
| 55 | *P. acuta* | 16:00 | 2.3 | . | 1 | KR919852 | 19 | 200 | 0.33 | 0.86 | 3.7 | 1 |
| 56 | *P. acuta* | 16:06 | 2.5 | . | 2 | KR919846 | 16 | 120 | 0.14 | 0.54 | **5.2** | 4 |
|  | **Fulaga avg.±std. dev.** | | 7.6±7.5 | NA | 1.3±0.5 |  | 17±6.1^ab^ | 170±140^ab^ | 0.18±0.12 | 0.68±0.28 | 3.3±0.2 | 1.1±1.7 |
| **Kabara** (4 analyzed/13 collected) | | | | | |  |  |  |  |  |  |  |
| 60 | *P. acuta* | 11:24 | 12.6 | 140 | 3 | KT381823 | 8 | 23 | 0.07 | 0.39 | 2.5 | 0 |
| 66 | *P. acuta* | 15:43 | 14.4 | 33 | 2 | KP942639 | 17 | 120 | 0.16 | 0.48 | 1.7 | 0 |
| 68 | *P. acuta* | 15:53 | 14.4 | 17 | 2 | KP942640 | 17 | 140 | 0.13 | 0.45 | **4.4** | 3 |
| 69 | *P. acuta* | 15:59 | 15.3 | 25 | 2 | KP942641 | 13 | 86 | 0.10 | 0.17 | 2.6 | 0 |
|  | **Kabara avg.±std. dev.** | | 14±1.1 | 54±58^ab^ | 2.3±0.5 |  | 14±4.3^ab^ | 92±51^ab^ | 0.12±0.04 | 0.37+0.14 | 2.8±1.1 | 0.8±1.5 |
| **Tuvuca** (8 analyzed/8 collected) | | | | | |  |  |  |  |  |  |  |
| 83 | *P. acuta* | 8:47 | 30.5 | . | 1 | KP942656 | 31 | 420 | 0.15 | 0.69 | MD | MD |
| 84 | *P. verrucosa* | 9:07 | 16.3 | 47 | 1 | KP942657 | 15 | 96 | 0.43 | 1.3 | 2.7 | 0 |
| 85 | *P. verrucosa* | 9:15 | 10.0 | 198 | 2 | KR919842 | 15 | 120 | 0.36 | 0.19 | MD | MD |
| 86 | *P. verrucosa* | 9:18 | 11.2 | 53 | 2 | KR919849 | 19 | 180 | 0.18 | 1.6 | 2.8 | 0 |
| 87 | *P. verrucosa* | 9:20 | 11.1 | 27 | 1 | KR919841 | 20 | 170 | 0.31 | 1.4 | 3.4 | 1 |
| 88 | *P. verrucosa* | 9:30 | 4.3 | 284 | 1 | KP942658 | 41 | 550 | **0.67** | + | MD | 1 |
| 89 | *P. acuta* | 11:15 | 25.5 | 173 | 1 | KP942659 | 15 | 110 | 0.48 | 0.80 | 1.7 | 0 |
| 90 | *P. acuta* | 11:18 | 28.0 | 74 | 1 | KP942660 | 16 | 150 | 0.16 | + | MD | MD |
|  | **Tuvuca avg.±std. dev.** | | 17±9.7 | 122±97^ab^ | 1.3±0.5 |  | 22±9.5^ab^ | 230±170^ab^ | 0.34±0.18 | 1.0±0.53 | 2.7±0.7 | 0.4±0.5 |
| **Cicia** (8 analyzed/12 collected) | | |  |  |  |  |  |  |  |  |  |  |
| 91 | . | 9:42 | 31.2 | 33 | 1 | . | 10 | 60 | + | + | MD | MD |
| 93 | *P. verrucosa* | 10:12 | 13.0 | 91 | 2 | KP942631 | 35 | 250 | 0.50 | 0.51 | 3.4 | 1 |
| 95 | *P. verrucosa* | 10:17 | 15.3 | 74 | 1 | KP942632 | % | % | 0.35 | + | MD | MD |
| 97 | *P. verrucosa* | 10:27 | 8.4 | 103 | 1 | KP942633 | 18 | 110 | 0.28 | 1.7 | MD | MD |
| 98 | *P. verrucosa* | 10:31 | 4.5 | 508 | 4 | KP942634 | 37 | 650 | 0.18 | + | MD | MD |
| 99 | Sampled healthy portion of previous colony | | | | 1 |  |  |  | 0.31 | **2.2** | 3.7 | 1 |
| 101 | *P. damicornis* | 9:20 | 4.0 | 118 | 1 | KR919855 | 11 | 88 | 0.12 | 0.64 | 1.6 | 0 |
| 102 | *P. verrucosa^#^* | 11:51 | 9.5 | 91 | 1 | KP942635 | 52 | 960 | 0.19 | 0.65 | MD | 0 |
|  | **Cicia avg.±std. dev.** | | 12±9.3 | 145±162^ab^ | 1.6±1.1 |  | 27±17^ab^ | 353±369^ab^ | 0.27±0.14 | 0.88±0.55 | 2.9±1.1 | 0.5±0.6 |
| **Mago** (9 analyzed/9 collected) | | |  |  |  |  |  |  |  |  |  |  |
| 103 | *P. brevicornis* | 8:34 | 3.0 | . | 3 | KR919858 | 10 | 33 | 0.39 | 0.25 | 2.1 | 0 |
| 104 | *P. verrucosa* | 10:57 | 9.5 | . | 1 | KR919853 | 7 | 29 | 0.17 | 1.1 | 2.2 | 0 |
| 105 | *P. damicornis* | 11:01 | 10.0 | . | 1 | KR092029 | 10 | 45 | 0.28 | 0.89 | 1.3 | 0 |
| 106 | *P. meandrina* | 11:07 | 6.6 | . | 1 | KR092030 | 11 | 77 | 0.30 | 0.71 | 3.0 | 0 |
| 107 | *P. meandrina* | 11:16 | 5.6 | . | 1 | KR092031 | 13 | 67 | 0.22 | 0.78 | 1.7 | 0 |
| 108 | *P. meandrina* | 11:21 | 6.6 | . | 2 | KR919851 | 7 | 28 | 0.26 | + | MD | 0 |
| 109 | *P. acuta* | 13:22 | 9.9 | . | 2 | KP942642 | 23 | 300 | 0.16 | 0.71 | MD | MD |
| 110 | *P. acuta* | 13:31 | 7.8 | . | 2 | KT156727 | 23 | 330 | 0.37 | 0.81 | 1.5 | 0 |
| 111 | *P. acuta* | 13:36 | 5.6 | . | 1 | KP942643 | 18 | 170 | 0.13 | 0.39 | **4.7** | 1 |
|  | **Mago avg.±std. dev.** | | 7.2±2.4 | NA | 1.6±0.7 |  | 14±6.3^b^ | 120±119^b^ | 0.25±0.09 | 0.71±0.27 | 2.4±1.2 | 0.13±0.35 |
| **Vanua Balavu** (19 analyzed/42 collected) | | | |  |  |  |  |  |  |  |  |  |
| 112 | *P. acuta* | 9.46 | 18.9 | 37 | 1 | KP942661 | 12 | 81 | 0.14 | 0.21 | 2.1 | 0 |
| 113 | *P. acuta* | 10:08 | 13.9 | 66 | 1 | KP942662 | 26 | 220 | 0.22 | 0.49 | 2.2 | 0 |
| 114 | . | 10:19 | 5.0 | 36 | 2 | . | * | * | 0.22 | + | MD | 0 |
| 115 | *P. damicornis* | 10:26 | 7.0 | 123 | 2 | KR919829 | 11 | 70 | 0.13 | 0.29 | **8.2** | 1 |
| 116 | *P. acuta* | 12:35 | 8.0 | 165 | 1 | KP942663 | 9 | 44 | 0.26 | 0.54 | 1.9 | 0 |
| 117 | *P. acuta* | 13:02 | 11.0 | 140 | 2 | KR092032 | 7 | 29 | 0.21 | 1.3 | 2.2 | 0 |
| 118 | *P. acuta* | 13:14 | 8.2 | 411 | 2 | KP942664 | 10 | 47 | 0.11 | 0.91 | 2.8 | 0 |
| 119 | *P. acuta^* | 9:35 | 27.1 | 17 | 1 | KP942665 | 12 | 86 | 0.52 | 0.55 | 3.8 | 0 |
| 120 | *P. acuta* | 9:40 | 25.5 | 41 | 1 | KP942666 | 22 | 230 | 0.49 | 0.48 | 2.8 | 0 |
| 123 | *P. acuta* | 10:03 | 8.7 | 174 | 1 | KP942667 | 19 | 170 | 0.44 | 0.29 | **5.8** | 2 |
| 124 | *P. acuta* | 10:20 | 6.0 | 154 | 1 | KR919847 | 16 | 86 | 0.29 | 0.49 | 2.9 | 0 |
| 130 | *P. damicornis* | 9:26 | 16.3 | 74 | 4 | KR919848 | 8 | 42 | 0.22 | 1.0 | 1.8 | 0 |
| 131 | *P. damicornis* | 9:41 | 10.9 | 49 | 1 | KP942668 | * | * | 0.55 | 0.86 | 2.3 | 0 |
| 134 | *P. acuta* | 11:26 | 31.7 | 49 | 1 | KT156728 | 17 | 140 | 0.14 | 1.1 | 2.0 | 0 |
| 138 | *P. acuta* | 16:10 | 8.5 | 8 | 1 | KP942669 | * | * | 0.29 | 0.96 | 2.2 | 0 |
| 146 | *P. acuta* | 9:28 | 9.6 | 140 | 1 | KP942670 | 17 | 170 | 0.04 | 0.18 | **8.0** | 2 |
| 147 | *P. acuta* | 9:32 | 9.9 | 82 | 4 | KP942671 | 15 | 140 | 0.04 | 0.32 | 2.7 | 0 |
| 151 | *P. acuta* | 11:47 | 27.4 | 58 | 3 | KP942672 | 20 | 170 | 0.15 | 1.4 | 3.3 | 0 |
| 153 | *P. acuta* | 14:50 | 21.1 | 49 | 1 | KP942673 | 12 | 71 | 0.11 | **1.7** | **5.5** | 3 |
| **Vanua Balavu avg.±std. dev.** | | | 14±8.3 | 99±92^b^ | 1.6±1.0 |  | 15±5.4^b^ | 110±65^b^ | 0.24±0.16 | 0.73±0.45 | 3.5±2.0 | 0.4±0.9 |
| **Lau Archipelago avg.±std. dev.** | | | 11±6.8 | 133±123 | 1.4±0.8 |  | 18±12 | 210±310 | 0.27±0.14 | 0.74±0.45 | **Outlier freq.** | **11/70 (16%)** |
| **Australs-Cooks avg.±std. dev.** | | | 14±3.1 | . | 2.2±0.9 |  | 8.5±3.5 | 47±39 | 0.27±0.12 | 0.55±0.37 | **Outlier freq.** | **5/45**  **(11%)** |

^+^DNA extraction failed. *no picture taken or no scaling object in picture. ^%^entire colony was not encompassed in image. ^hosts *Symbiodinium* of clades A and C. ^$^was determined to be a Mahalanobis distance outlier upon imputing data (see explanation in main text.). ^#^hosts *Symbiodinium* of clades C and D.
