# Supplementary material for: Identifying corals displaying aberrant behavior in Fiji’s Lau Archipelago
Source: PLoS One. 2017 May 24;12(5):e0177267. doi: 10.1371/journal.pone.0177267 (PMC5443480; doi:10.1371/journal.pone.0177267)
Supplement: S2 Table — All assays utilized SYBR® Green chemistry except for the Solaris™ RNA spike, which required the use of a proprietary Taqman® probe provided by the manufacturer (described in the Methods A in S1 File). Corals characterized by either highly elevated or severely diminished expression levels of the target genes were hypothesized to be displaying aberrant behavior at the time of sampling (see the main text for details.). bp = base pairs. *p<0.05. **p<0.001. (DOCX) [file pone.0177267.s003.docx]

**S2 table. Target genes and real-time PCR conditions**. All assays utilized SYBR® Green chemistry except for the Solaris™ RNA spike, which required the use of a proprietary Taqman® probe provided by the manufacturer (described in the S1 methods). Corals characterized by either highly elevated or severely diminished expression levels of the target genes were hypothesized to be displaying aberrant behavior at the time of sampling (see the main text for details.). bp=base pairs. **p*<0.05. ***p*<0.001.

| **Gene name** | **Abbre-viation** | **function** | **compartment** | **amplicon length** (bp) | **[primer]** (nM) | **anneal. temp.** (ºC) | **Cy-cle #** | **Primary finding** |
| --- | --- | --- | --- | --- | --- | --- | --- | --- |
| Solaris RNA spike | spike | spike | exogenous | proprie-tary | 1X | 60 | 40 | Significant variation between 96-well plates** |
| ribulose-1,5-bisphosphate  carboxylase/oxygenase | *rbcL* | photosynthe-sis^a^ | *Symbiodinium* | 126 | 200 | 60 | 35 | 3-fold higher expression in outliers** |
| zinc-induced facilitator-like 1-like | *zifl1l* | metabolism^b^ | *Symbiodinium* | 69 | 500 | 60 | 35 | 4-fold higher expression in outliers* |
| heat shock protein 90 | *hsp90* | stress^c^ | *Symbiodinium* | 89 | 500 | 60 | 35 | 4-fold higher expression in outliers** |
| ubiquitin ligase | *ubiq-lig* | stress^c^ | *Symbiodinium* | 122 | 300 | 60 | 40 | 3.5-fold higher expression in outliers** |
| ascorbate peroxidase | *apx1* | stress^a^ | *Symbiodinium* | 107 | 150 | 61 | 40 | 2-fold higher expression in outliers |
| carbonic anhydrase | *ca* | metabolism^b^ | host | 122 | 250 | 60 | 30 | Significant variation between sites* |
| lectin | *lectin* | cell adhesion^b^ | host | 147 | 300 | 61 | 40 |  |
| copper-zinc superoxide dismutase | *cu-zn-sod* | stress^c^ | host | 79 | 100 | 60 | 32 | 3-fold higher expression in outliers** |
| green fluorescent protein-like  chromoprotein | *gfp-cp* | various^c,d^ | host | 199 | 500 | 59 | 35 | 3-fold higher expression in outliers* |

^a^[9]. ^b^[10]. ^c^[5]. ^d^see main text for hypothetical function.
