# Supplementary material for: Identifying corals displaying aberrant behavior in Fiji’s Lau Archipelago
Source: PLoS One. 2017 May 24;12(5):e0177267. doi: 10.1371/journal.pone.0177267 (PMC5443480; doi:10.1371/journal.pone.0177267)
Supplement: S3 Table — To calculate the principal component (PC) score (first axis only; PC1), principal components analysis (PCA) was performed on the 11 molecular-scale response variables only (i.e., excluding size data, but including biological composition data [RNA/DNA ratio and Symbiodinium genome copy proportion (GCP)]). The global mean r2 between the PC1 score and the Mahalanobis distance was 0.40, and this positive correlation was statistically significant (linear regression t-test, p<0.001). When a sample was considered an outlier, the value(s) for the response variable(s) that had Z-scores <-2 or >2 has/have been highlighted in bold font. Frequencies (freq.) in the “Outlier?” column represent the number of outliers over the total number of samples for the respective island with enough data to calculate the Mahalanobis distance; please note that this may be lower than the total number of samples analyzed for that island. When gene expression varied significantly across islands within the Lau Archipelago (see Table 2 for ANOVAs conducted with normalized data.), the gene name has been highlighted in bold font, and for cu-zn-sod, Tukey’s honestly significant differences were detected (p<0.05; denoted by lower-case letters). When a significant difference (student’s t-test, p<0.05) was detected between the two regions (Lau Province, Fiji vs. Austral Islands, French Polynesia+Cook Islands [11]), the lower of the two means has been underlined. The “Maximum/minimum” fold difference value in the final row was calculated by dividing the highest expression level of the dataset by the lowest. Ct = threshold cycle. “.” = missing data. MD = value could not be calculated due to “missing data.” NA = not assessed. (DOCX) [file pone.0177267.s004.docx]

**S3 table. Sample information II-gene expression data**. To calculate the principal component (PC) score (first axis only; PC1), principal components analysis (PCA) was performed on the 11 molecular-scale response variables only (i.e., excluding size data, but including biological composition data [RNA/DNA ratio and *Symbiodinium* genome copy proportion (GCP)]). The global mean *r*^2^ between the PC1 score and the Mahalanobis distance was 0.40, and this positive correlation was statistically significant (linear regression *t*-test, *p*<0.001). When a sample was considered an outlier (highlighted in blue), the value(s) for the response variable(s) that was/were characterized by *Z*-score(s) <-2 or >2 has/have been highlighted in bold font. Frequencies (freq.) in the “Outlier?” column represent the number of outliers over the total number of samples for the respective island with enough data to calculate the Mahalanobis distance; please note that this may be lower than the total number of samples analyzed for that island (S1 table). When gene expression varied significantly across islands within the Lau Archipelago (see Table 2 for ANOVAs conducted with normalized data.), the gene name has been highlighted in bold font, and for *cu-zn-sod*, Tukey’s honestly significant differences were detected (*p*<0.05; denoted by lower-case letters). When a significant difference (student’s *t*-test, *p*<0.05) was detected between the two regions (Lau Province, Fiji vs. Austral Islands, French Polynesia+Cook Islands [11]), the lower of the two means has been underlined. The “Maximum/minimum” fold difference value in the final row was calculated by dividing the highest expression level of the dataset by the lowest. Ct=threshold cycle. “.”=missing data. MD=value could not be calculated due to “missing data.” NA=not assessed.

| **Island** | Host | ***Symbiodinium* mRNAs**  (non-normalized, inverse Ct values x 10^9^) | | | | | **Host coral mRNAs**  (non-normalized, inverse Ct values x 10^9^) | | | | PC1 score | Outlier? |
| --- | --- | --- | --- | --- | --- | --- | --- | --- | --- | --- | --- | --- |
| Sample | | *rbcL* | *zifl1l* | *hsp90* | *ubiq-lig* | *apx1* | *ca* | *lectin* | ***cu-zn-sod*** | ***gfp-cp*** |  |  |
| **Totoya** (13 analyzed/20 collected) | | | | | |  |  |  |  |  |  |  |
| 2 | . | 78.6 | 8.17 | 10.8 | 0.792 | 1.48 | 29.8 | 3.99 | 2.96 | 12.7 | ^#^ | ^#^ |
| 3 | *P. damicornis* | 0.329 | 0.183 | 0.731 | 0.025 | . | . | 1.78 | 0.241 | 131 | MD | MD |
| 4 | *P. damicornis* | 125 | 0.869 | 66.9 | 2.14 | 21.6 | 13.9 | 44.1 | 0.614 | 304 | 0.88 | no |
| 5 | *P. damicornis* | 35.9 | 5.27 | 13.0 | 0.241 | 3.17 | 238 | 3.40 | 10.5 | 78.6 | -1.3 | no |
| 6 | *P. damicornis* | 24.2 | 0.756 | 19.2 | 0.420 | 4.38 | 9.50 | 1.91 | 1.93 | 48.4 | 0.17 | no |
| 7 | *P. damicornis* | 181 | 84.3 | 31.9 | 1.58 | 6.64 | 68.5 | 10.1 | 10.1 | **2890** | 0.52 | yes |
| 9 | . | 287 | 1.62 | 40.2 | 2.24 | 6.19 | 337 | 21.1 | 14.9 | 2000 | NA^#^ | MD |
| 10 | *P. damicornis* | 353 | 1.55 | 80.5 | 3.17 | 15.6 | 274 | **78.6** | 20.1 | 1020 | 0.31 | no |
| 15 | *P. damicornis* | 42.1 | 58.2 | 43.1 | 0.869 | 3.73 | 55.6 | **68.5** | 8.96 | **1950** | -0.19 | yes |
| 16 | *P. meandrina* | 81.4 | 29.1 | 128 | 1.26 | 7.45 | 477 | 10.5 | 19.2 | 274 | -1.0 | no |
| 17 | *P. meandrina* | 587 | 56.9 | 107 | 5.09 | 23.7 | 2000 | 21.8 | 42.1 | 61.7 | -1.0 | no |
| 19 | *P. meandrina* | 256 | 14.9 | 66.9 | 1.15 | 5.65 | MD | 4.59 | 26.6 | 1320 | MD | MD |
| 20 | *P. damicornis* | 56.9 | 27.2 | 84.3 | 1.12 | 5.39 | 396 | 8.76 | 13.3 | 140 | -1.9 | no |
| **Totoya avg.**±**std. dev.** | | 162±169 | 22.2±27.7 | 53.3±39.4 | 1.55±1.38 | 8.75±7.37 | 354±571 | 21.5±26.0 | 13.2±11.9^b^ | 787±962 | **Freq: 2/9** | |
| **Matuku** (10 analyzed/10 collected) | | | | | |  |  |  |  |  |  |  |
| 21 | *P. verrucosa* | 125 | 19.7 | 132 | 2.22 | 9.17 | 137 | 84.3 | 10.2 | 131 | 0.47 | no |
| 22 | *P. verrucosa* | 143 | 30.5 | 114 | 2.70 | 7.45 | 378 | 46.8 | 17.9 | 59.6 | -1.9 | no |
| 23 | *P. verrucosa* | 125 | 1.36 | 58.2 | 2.14 | 6.49 | 73.4 | 66.9 | 9.83 | 208 | -0.1 | no |
| 24 | *P. damicornis* | 1290 | 353 | 122 | 9.39 | 21.1 | 157 | 111 | 25.9 | 47.3 | 0.10 | no |
| 25 | *P. verrucosa* | **326** | 29.8 | 66.9 | 1.74 | 13.6 | 61.0 | 40.2 | 5.85 | 101 | 3.6 | yes |
| 26 | *P. meandrina* | 307 | 415 | 84.3 | 3.17 | 4.18 | 3640 | 6.49 | 128 | 1660 | -1.2 | no |
| 27 | *P. damicornis* | 61.7 | 134 | 82.4 | 0.998 | 3.48 | 150 | 4.69 | 26.6 | **3440** | -0.4 | no |
| 28 | *P. damicornis* | 96.8 | 189 | 58.2 | 1.32 | 7.62 | 548 | 24.8 | 30.5 | 1046 | -1.5 | no |
| 29 | *P. verrucosa* | 1170 | 238 | 111 | 7.99 | . | 1.41 | 11.3 | 22.1 | 4.80 | MD | MD |
| 30 | *P. damicornis* | 45.2 | 59.6 | 56.9 | 0.889 | 2.46 | 140 | 44.1 | 14.9 | 1290 | -0.92 | no |
| **Matuku** **avg.**±**std. dev.** | | 369±464 | 147±148 | 88.6±28.9 | 3.26±2.97 | 8.39±5.82 | 529±1105 | 44.1±35.0 | 29.2±35.7^ab^ | 799±1110 | **Freq: 1/9** | |
| **Moala** (12 analyzed/17 collected) | | | | | |  |  |  |  |  |  |  |
| 33 | *P. verrucosa* | 86.3 | 1.80 | 49.5 | 2.29 | 4.59 | 345 | 43.1 | 12.4 | 55.6 | -1.6 | no |
| 34 | *P. meandrina* | 2000 | 2.76 | 165 | 12.4 | 19.7 | 177 | 128 | 16.3 | 125 | 0.16 | no |
| 35 | . | 523 | 2.96 | 70.1 | 3.10 | 5.78 | 9.17 | 7.11 | 12.1 | 2.76 | MD^#^ | MD^#^ |
| 37 | *P. verrucosa* | 954 | 59.6 | 90.3 | 8.76 | 21.6 | 5390 | 23.7 | 48.4 | 128 | -1.6 | no |
| 38 | . | 315 | 31.9 | 50.7 | 2.96 | 7.27 | 511 | 43.1 | 8.56 | 40.2 | MD^#^ | MD^#^ |
| 39 | *P. verrucosa* | 999 | 22.6 | 131 | 6.79 | 16.7 | 775 | **198** | 14.2 | 88.3 | 0.48 | yes |
| 40 | *P. verrucosa* | 122 | 9.39 | 140 | 2.46 | 5.91 | 33.1 | 38.4 | 8.17 | 185 | 0.27 | no |
| 41 | *P. verrucosa* | 35.4 | 4.18 | 45.2 | 0.477 | 2.89 | 14.9 | 21.6 | 2.14 | 84.3 | -0.89 | no |
| 42 | *P. verrucosa* | 723 | 3.17 | 84.3 | 4.69 | 12.1 | 212 | 4.18 | 6.71 | 40.2 | -0.28 | no |
| 44 | *P. acuta* | 1120 | 6.64 | 161 | 9.39 | 29.1 | 345 | 29.8 | 80.5 | 2190 | -1.2 | no |
| 45 | *P. verrucosa* | 1480 | 228 | 154 | 9.39 | 10.1 | 1550 | 238 | 23.1 | 42.1 | -0.49 | no |
| 47 | *P. verrucosa* | 1590 | 189 | 111 | 8.76 | 26.6 | 954 | 45.2 | 50.7 | 2240 | -0.46 | no |
| **Moala avg.**±**std. dev.** | | 829±643 | 46.8±77.8 | 104±45.3 | 5.96±3.77 | 13.5±9.00 | 860±1500 | 68.3±77.1 | 23.6±23.7^ab^ | 435±833 | **Freq: 1/10** | |
| **Fulaga** (7 analyzed/9 collected) | | | | | |  |  |  |  |  |  |  |
| 48 | *P. acuta* | 976 | 329 | 76.9 | 6.49 | 2.46 | 548 | 34.2 | 43.6 | 53.1 | 1.6 | no |
| 49 | *P. damicornis* | 2350 | 61.0 | 185 | 13.0 | 9.39 | 1260 | 147 | 65.4 | 2240 | 0.67 | no |
| 50 | *P. damicornis* | 0.535 | 0.0569 | 1.19 | 0.0179 | 0.0127 | 13.9 | 2.14 | 0.830 | 9.17 | -1.7 | no |
| 51 | *P. acuta* | 88.3 | 56.9 | 27.8 | 0.238 | 3.03 | 629 | 3.40 | 20.1 | 99.1 | -0.95 | no |
| 54 | *P. acuta* | **1022** | 6.49 | 116 | **5.65** | **20.6** | 659 | 9.39 | 84.3 | 300 | MD | * |
| 55 | *P. acuta* | 99.1 | 9.83 | 71.7 | 1.35 | 2.46 | 212 | 5.03 | **92.5** | 574 | -0.88 | no |
| 56 | *P. acuta* | **337** | 18.3 | **122** | **3.40** | 9.39 | 157 | 8.36 | **61.0** | 353 | 5.8 | yes |
| **Fulaga avg.**±**std. dev.** | | 696±842 | 68.8±117 | 85.8±61.7 | 4.31±4.59 | 6.76±7.09 | 497±420 | 29.9±52.8 | 52.5±33.3^ab^ | 518±785 | **Freq: 1/6** | |
| **Kabara** (4 analyzed/13 collected) | | | | | |  |  |  |  |  |  |  |
| 60 | *P. acuta* | 5.03 | 0.869 | 9.50 | 0.0924 | 0.228 | 11.8 | 0.998 | 4.09 | 143 | -0.69 | no |
| 66 | *P. acuta* | 523 | 3.81 | 114 | 5.39 | 4.80 | 1780 | 43.6 | 94.6 | 370 | -0.10 | no |
| 68 | *P. acuta* | 244 | 8.36 | **88.3** | **2.40** | 5.91 | 21.1 | 55.6 | **65.4** | 287 | 4.4 | yes |
| 69 | *P. acuta* | 5.15 | 0.109 | 3.99 | 0.0862 | 0.488 | 212 | 5.27 | 12.1 | 23.7 | -1.2 | no |
| **Kabara avg.**±**std. dev.** | | 194±246 | 3.29±3.74 | 53.9±55.6 | 1.99±2.51 | 2.86±2.92 | 506±854 | 26.4±27.3 | 44.0±43.0^ab^ | 206±153 | **Freq: 1/4** | |
| **Tuvuca** (8 analyzed/8 collected) | | | | | |  |  |  |  |  |  |  |
| 83 | *P. acuta* | 1046 | 173 | 217 | 9.17 | MD | 548 | 88.3 | 71.7 | 86.3 | MD | MD |
| 84 | *P. verrucosa* | 1907 | 250 | 217 | 13.0 | 22.1 | 587 | 23.1 | 25.1 | 256 | -0.73 | no |
| 85 | *P. verrucosa* | 48.4 | 20.6 | 35.9 | 0.830 | MD | 999 | 5.85 | 23.1 | 523 | MD | MD |
| 86 | *P. verrucosa* | 244 | 34.2 | 71.7 | 1.48 | 8.56 | 911 | 70.1 | 11.3 | 307 | -0.85 | no |
| 87 | *P. verrucosa* | 22.6 | 2.70 | 42.1 | 0.488 | 2.76 | 12.1 | **24.8** | 1.48 | 16.3 | -0.55 | no |
| 88 | *P. verrucosa* | 353 | 228 | 96.8 | 5.15 | 5.65 | 1046 | 2.63 | 35.9 | 869 | MD | MD |
| 89 | *P. acuta* | 250 | 48.4 | 51.9 | 2.40 | 8.36 | 345 | 76.0 | 19.2 | 499 | -1.6 | no |
| 90 | *P. acuta* | 445 | 11.6 | 90.3 | 5.03 | 14.9 | 1046 | 19.2 | 48.4 | 163 | MD | MD |
| **Tuvuca avg.**±**std. dev.** | | 540±638 | 96.1±103 | 103±73.7 | 4.69±4.43 | 10.4±7.01 | 687±379 | 38.7±33.9 | 29.5±22.2^ab^ | 340±280 | **Freq: 0/4** | |
| **Cicia** (8 analyzed/12 collected) | | |  |  |  |  |  |  |  |  |  |  |
| 91 | . | 208 | 6.49 | 40.7 | 2.09 | 4.80 | 37.5 | 1.46 | 4.92 | 25.9 | MD^#^ | MD^#^ |
| 93 | *P. verrucosa* | 238 | 181 | 55.6 | 2.82 | 3.90 | **2820** | 88.3 | 48.4 | 181 | -2.2 | no |
| 95 | *P. verrucosa* | 345 | 76.9 | 68.5 | 5.52 | 7.45 | 2290 | 21.6 | 21.6 | 128 | MD | MD |
| 97 | *P. verrucosa* | 3.99 | 0.161 | 1.23 | 0.0291 | 0.217 | 3.17 | 1.46 | MD | 1.15 | MD^@^ | MD |
| 98 | *P. verrucosa* | 47.3 | 208 | 10.3 | 0.774 | 1.62 | 4088 | 125 | 51.9 | 96.8 | MD | MD |
| 99 | Same as 98 | 1046 | 189 | 131 | 6.34 | 17.9 | 2403 | 238 | 21.1 | 26.6 | -0.69 | no |
| 101 | *P. damicornis* | 548 | 80.5 | 101 | 3.90 | 10.8 | 134 | 3.86 | 42.1 | 33.5 | 0.64 | no |
| 102 | *P. verrucosa*^%^ | 830 | 34.2 | 99.1 | 4.69 | MD | 425 | 61.0 | 15.6 | 37.5 | MD | MD |
| **Cicia avg.**±**std. dev.** | | 408±372 | 97.0±84.5 | 63.4±45.6 | 3.27±2.24 | 6.67±6.08 | 1525±1571 | 67.6±82.6 | 29.4±18.0^ab^ | 66.3±62.4 | **Freq: 0/3** | |
| **Mago** (9 analyzed/9 collected) | | |  |  |  |  |  |  |  |  |  |  |
| 103 | *P. brevicornis* | 71.7 | 361 | 62.4 | 1.82 | 6.64 | 455 | 17.1 | 27.2 | 345 | -1.1 | no |
| 104 | *P. verrucosa* | 315 | 71.7 | 94.6 | 3.99 | 14.6 | 999 | 25.9 | 13.9 | 150 | -0.19 | no |
| 105 | *P. damicornis* | 869 | 140 | 99.1 | 5.65 | 19.7 | 1700 | 94.6 | 54.3 | 425 | -0.27 | no |
| 106 | *P. meandrina* | 181 | 111 | 31.2 | 0.811 | 4.69 | 629 | 6.27 | 76.9 | 890 | -0.89 | no |
| 107 | *P. meandrina* | 561 | 104 | 99.1 | 3.32 | 21.6 | 137 | 12.1 | 35.0 | 96.8 | 0.56 | no |
| 108 | *P. meandrina* | 361 | 109 | 76.9 | 3.10 | 5.91 | 1150 | 3.24 | 49.5 | 50.7 | MD | MD |
| 109 | *P. acuta* | MD | 54.3 | 66.9 | 1.78 | MD | 189 | 90.3 | 33.5 | 361 | MD | MD |
| 110 | *P. acuta* | 88.3 | 15.2 | 31.2 | 0.889 | 1.91 | 561 | 16.5 | 40.2 | 59.6 | -1.7 | no |
| 111 | *P. acuta* | 488 | 49.5 | 73.4 | **4.80** | 14.6 | 116 | 12.4 | 82.4 | 378 | 3.8 | yes |
| **Mago avg.**±**std. dev.** | | 367±269 | 113±101 | 70.5±26.1 | 2.91±1.71 | 11.2±7.38 | 660±534 | 30.9±35.5 | 45.9±22.5^ab^ | 306±264 | **Freq: 1/7** | |
| **Vanua Balavu** (19 analyzed/42 collected) | | |  |  |  |  |  |  |  |  |  |  |
| 112 | *P. acuta* | 322 | 104 | 65.4 | 3.90 | 5.27 | 1907 | 46.2 | 41.2 | 228 | -0.54 | no |
| 113 | *P. acuta* | 757 | 811 | 165 | 6.05 | 3.32 | 674 | 6.71 | 125 | 615 | 0.055 | no |
| 114 | . | 644 | 58.2 | 96.8 | 5.78 | 5.91 | 1170 | 96.8 | 58.2 | 147 | MD | MD |
| 115 | *P. damicornis* | 238 | **706** | 99.1 | 2.40 | 6.19 | 1202 | 22.6 | 68.5 | 523 | 2.2 | yes |
| 116 | *P. acuta* | 1046 | 37.5 | 109 | 5.65 | 7.99 | 811 | 70.1 | 59.6 | 307 | **-**0.005 | no |
| 117 | *P. acuta* | 177 | 41.2 | 29.1 | 1.15 | 3.17 | 723 | 16.0 | 35.9 | 233 | -1.0 | no |
| 118 | *P. acuta* | 265 | 73.4 | 51.9 | 3.48 | 6.34 | 1130 | 30.9 | 59.6 | 2350 | 0.68 | no |
| 119 | *P. acuta^* | 615 | 8.36 | 185 | 3.56 | 11.8 | 38.4 | 28.5 | 27.8 | 109 | 0.34 | no |
| 120 | *P. acuta* | 326 | 6.49 | 104 | 2.54 | 8.96 | 12.1 | 35.0 | 15.6 | 106 | -0.24 | no |
| 123 | *P. acuta* | 287 | 56.9 | 194 | 4.13 | 20.6 | **1170** | 53.7 | **125** | 222 | -0.23 | yes |
| 124 | *P. acuta* | 81.4 | 65.4 | 55.6 | 0.783 | 6.34 | 14.9 | 13.4 | 4.38 | 256 | 1.3 | no |
| 130 | *P. damicornis* | 106 | 16.0 | 27.8 | 1.38 | 2.52 | 523 | 17.1 | 6.95 | 187 | -1.4 | no |
| 131 | *P. damicornis* | 548 | 274 | 99.1 | 6.05 | 10.8 | 1380 | 61.0 | 99.1 | 477 | -1.7 | no |
| 134 | *P. acuta* | 911 | 150 | 244 | 6.95 | 16.3 | 122 | 84.3 | 53.1 | 165 | 1.8 | no |
| 138 | *P. acuta* | 890 | 2.82 | 222 | 9.39 | 17.1 | 345 | 96.8 | 90.3 | 2700 | 0.93 | no |
| 146 | *P. acuta* | 62.4 | 8.36 | **75.1** | **0.830** | 4.92 | 38.4 | 2.19 | 16.7 | 40.2 | 6.1 | yes |
| 147 | *P. acuta* | 18.8 | 44.1 | 6.05 | 0.185 | 0.774 | 644 | 16.3 | 22.6 | 294 | -0.51 | no |
| 151 | *P. acuta* | 811 | 24.8 | 101 | 3.81 | 14.9 | 706 | 150 | 61.0 | 811 | 1.6 | no |
| 153 | *P. acuta* | **256** | 7.99 | 37.5 | 1.62 | 6.19 | **1350** | 12.4 | 50.7 | 511 | 2.2 | yes |
| **Vanua Balavu avg.**±**std. dev.** | | 440±325 | 131±231 | 104±68.7 | 3.67±2.48 | 8.39±5.47 | 735±554 | 45.3±39.3 | 53.7±36.1^a^ | 541±729 | **Freq: 4/18** | |
| **Lau Archipelago avg.**±**std. dev.** | | 453±491 | 87.5±138 | 84.2±54.0 | 3.57±3.11 | 9.04±6.95 | 711±935 | 42.7±49.7 | 35.6±30.8 | 491±739 |  |  |
| **Australs-Cooks avg.**±**std. dev.** | | . | . | 38.6±27.7 | 4.91±4.37 | 5.70±4.80 | . | . | . | . |  |  |
| **Maximum/minimum**  (fold difference) | | 7,000 | 14,000 | 300 | 700 | 2,000 | 4,000 | 200 | 500 | 3,000 |  |  |

^#^Data could not be normalized properly due to failed DNA extraction. *colony is likely an outlier, though the RNA/DNA ratio was not calculated due to the poor efficiency of the DNA extraction. ^%^hosts *Symbiodinium* of clades C and D. ^hosts *Symbiodinium* of clades A and C. ^@^cDNA performed poorly in qPCRs; reverse transcription reaction likely failed.
