# Supplementary material for: Identifying corals displaying aberrant behavior in Fiji’s Lau Archipelago
Source: PLoS One. 2017 May 24;12(5):e0177267. doi: 10.1371/journal.pone.0177267 (PMC5443480; doi:10.1371/journal.pone.0177267)
Supplement: S4 Table — Site and date were excluded from the analysis due to typically having too few samples (TFS) for a robust comparison. Unlike in the univariate comparisons pooled across species (Table 2), color was included in this species-specific analysis (n = 12 environmental parameters). Photosynthetically active radiation (n = 4 categorical groupings) was included in the analysis but excluded from the table, as it did not significantly affected any response variable for any of the three species. Likewise, only molecular physiological response variables (MPRV) for which at least one environmental parameter (EP) led to a significant difference (non-Bonferroni-adjusted) have been included in the table; as an exception, the multivariate centroid results have been included even when all were negative for a particular species (e.g., P. damicornis and P. verrucosa). Since 13 response variables were assessed across three species and 12 environmental parameters, 468 ANOVAs were performed; therefore a Bonferroni adjustment of 22 was made to the α level of 0.05, resulting in a multiple comparisons-adjusted α of 0.002; few results were statistically significant at this level, and those that were have been highlighted in green. The island number is listed as “variable” since not all species were found at each island. NS = not significant. NA = not applicable. ALCC = average live coral cover. (DOCX) [file pone.0177267.s005.docx]

**S4 table. Univariate and multivariate ANOVAs (MANOVAs) of the *Pocillopora acuta*, *P. damicornis*, and *P. verrucosa* datasets.** Site and date were excluded from the analysis due to typically having too few samples (TFS) for a robust comparison, though, unlike in the univariate comparisons pooled across species (Table 2), color *was* included in this species-specific analysis (n=12 environmental parameters). Photosynthetically active radiation (n=4 categorical groupings) was included in the analysis but excluded from the table, as it did not significantly affected any response variable for any of the three species. Likewise, only molecular physiological response variables (MPRV) for which at least one environmental parameter (EP) led to a significant difference (non-Bonferroni-adjusted) have been included in the table; as an exception, the multivariate centroid results have been included even when all were negative for a particular species (e.g., *P. damicornis* and *P. verrucosa*). Since 13 response variables were assessed across three species and 12 environmental parameters, 468 ANOVAs were performed; therefore, a Bonferroni adjustment of 22 was made to the α level of 0.05, resulting in a multiple comparisons-adjusted α of 0.002; few results were statistically significant at this level, and those that were have been highlighted in green. The island number is listed as “variable” since not all species were found at each island. NS=not significant. NA=not applicable. ALCC=average live coral cover.

| **EP (top row)/**  **MPRV (left-most column)** | island (variable) | exposure (n=3) | reef zone (n=4) | reef type (n=4) | time (n=3) | depth (n=7) | temp. (n=2) | salinity (n=9) | ALCC (n=5) | *Symbiodinium* assemblage (n=3) | color (n=4) |
| --- | --- | --- | --- | --- | --- | --- | --- | --- | --- | --- | --- |
| ***P. acuta*** (n=26 analyzed for all 13 MPRV) | | | | |  |  |  |  |  |  |  |
| Sym GCP | NS | *p*<0.05 | NS | NS | NS | NS | *p*<0.05 | NS | NS | *p*=0.05 | NS |
| RNA/DNA+ | NS | NS | NS | NS | NS | *p*<0.05 | NS | NS | NS | NS | NS |
| Sym *zifl1l*+ | NS | NS | NS | NS | *p*<0.05 | NS | NS | NS | *p*<0.01 | NS | NS |
| host *lectin*# | NS | *p*<0.05 | NS | NS | NS | NS | NS | NS | NS | NS | NS |
| host *gfp-cp*+ | NS | NS | NS | NS | NS | *p*<0.05 | NS | NS | NS | NS | NS |
| multivariate centroid | *p*<0.05 | *p*<0.05 | *p*<0.05 | *p*<0.05 | NS | NS | NS | NS | *p*<0.05 | NS | NS |
| ***P. damicornis*** (n=19 analyzed for all 13 MPRV) | | | |  |  |  |  |  |  |  |  |
| Sym *apx1*# | NS | NS | NS | NS | NS | NS | NS | *p*<0.05 | NS | NA (clade C only) | NS |
| Sym *hsp90*+ | NS | *p*<0.05 | NS | NS | NS | NS | NS | NS | NS | NA (clade C only) | NS |
| Sym *rbcL*# | NS | NS | NS | NS | NS | NS | NS | *p*<0.05 | NS | NA (clade C only) | NS |
| host *lectin*# | NS | NS | NS | *p*<0.05 | NS | NS | NS | NS | NS | NA (clade C only) | NS |
| host *gfp-cp*+ | *p*<0.05 | *p*<0.05 | NS | *p*<0.05 | NS | NS | NS | NS | NS | NA (clade C only) | NS |
| multivariate centroid | TFS | NS | NS | NS | NS | TFS | NS | TFS | NS | NA (clade C only) | NS |
| ***P. verrucosa*** (n=18 analyzed for all 13 MPRV) | | | |  |  |  |  |  |  |  |  |
| max. length# | *p*<0.05 | *p*<0.05 | NS | NS | NS | *p*<0.001 | NS | NS | NS | NA (clade C only) | NS |
| planar SA# | *p*<0.05 | NS | NS | NS | NS | *p*<0.01 | NS | NS | NS | NA (clade C only) | NS |
| Sym *ubiq-lig*# | NS | *p*<0.05 | NS | NS | NS | NS | NS | NS | NS | NA (clade C only) | *p*<0.05 |
| Sym *zifl1l*+ | NS | NS | NS | NS | *p*=0.001 | NS | NS | *p*<0.05 | *p*=0.002 | NA (clade C only) | NS |
| Sym *rbcL*# | NS | *p*<0.05 | NS | NS | NS | NS | NS | NS | NS | NA (clade C only) | NS |
| multivariate centroid | NS | NS | NS | NS | NS | NS | NS | NS | NS | NS | NS |

+log-transformed data. #rank-transformed data.
