# Supplementary material for: Identifying corals displaying aberrant behavior in Fiji’s Lau Archipelago
Source: PLoS One. 2017 May 24;12(5):e0177267. doi: 10.1371/journal.pone.0177267 (PMC5443480; doi:10.1371/journal.pone.0177267)
Supplement: S1 File — (DOCX) [file pone.0177267.s006.docx]

**SUPPORTING INFORMATION**

**Methods A**

**Satellite imagery and mapping**

Prior to departure, 1,704 km^2^ of WorldView2 (8-band) satellite imagery were acquired. The satellite images had a spatial resolution of 2 by 2-m (i.e., each pixel covered a 4 m^2^ area.), enabling real-time navigation in the field for locating structures of interest and avoiding emergent reefs. Multispectral satellite imagery data obtained from the DigitalGlobe WorldView2 satellite were used to create high-resolution bathymetric maps and habitat maps for shallow coral communities (*sensu* Saul and Purkis [1]). Briefly, groundtruthing efforts necessary to create such maps additionally required 1) aerial surveys of each island’s coastline and adjacent shallow marine habitats, 2) continuous bathymetry measures, 3) drop camera footage (787 “drop cam” events were conducted.), 4) characterization of sediment and hard substrates and habitat features using two acoustic, sub-bottom profiling techniques (Stratabox and Hydrobox), and 5) fine-scale photo-transect surveys. All maps and drop cam video footage will become publically available in mid-2017 on the following website: <http://maps.lof.org/lof>.

**Coral reef surveys**

A conductivity-temperature-depth (CTD) device (Castaway®, SonTek) was used to measure site temperature, salinity, and depth. Benthic surveys were performed by multiple divers (typically 2-5) at a range of depths (typically 5, 10, 15, 20, 25, and 30 m) using the point-intercept method at 10-cm intervals along a 10-m transect (100 points/transect). Cover of major functional groups (corals identified to genus, sponges, other invertebrates, and six groups of algae: macroalgae, crustose coralline algae [CCA], erect coralline algae, fine turfs, turf algae with sediment, and cyanobacteria) and substrate type (hardground, sand, mud, rubble, recently dead coral, bleached coral, or live coral) was quantified. In total 420 benthic transects were performed across the 70 sites, with an additional 198 transects performed to determine coral diversity (data not shown). Finally, large, motile invertebrates (urchins, octopus, lobsters, large crabs, large gastropods, and sea cucumbers) were identified and counted along coral belt transects and benthic point-intercept surveys, and one or two divers conducted timed swims at different depths to document the species diversity and abundance of sea cucumbers at each site. These data will be presented in a sister work.

**Pocilloporid coral sampling**

Corals were sampled with bone-cutting pliers at a range of depths, typically those at which benthic surveys were conducted (5, 10, 15, 20, 25, and 30 m, if possible). Pocilloporids were sampled by random swimming in the relative vicinity of the aforementioned transects, and there was always at least a 10-m distance between sampled colonies to avoid sampling clone-mates. When colonies of the α genotype of *P. damicornis* were not found, morphologically similar congenerics (e.g., *P. acuta*) were instead sampled. Approximately 50 mg of tissue and skeleton were removed from each colony, and the depth and time of coral collection were documented with a dive computer (Suunto). Photosynthetically active radiation (PAR) was measured next to each colony at the time of sampling as described in the main text. A scaling object of known length was placed next to each colony upon photographing it, and ImageJ (National Institutes of Health, USA) was used to estimate the maximum (max.) length (cm) and planar surface area (SA; cm^2^) of each colony *in silico*. A subjective estimate of the colony’s color was made *in situ* and verified by analysis of the images with ImageJ; scores of 1 (healthy), 2 (pale), 3 (very pale), or 4 (bleached) were given to each of the 153 colonies sampled across 46 of the 70 surveyed sites.

After the dive, coral fragments were immersed in RNALater® (Life Technologies, USA) on a small craft vessel and frozen at -20ºC for several hours. Once aboard the main ship, samples were homogenized in a mortar and pestle in RNAlater, and the tissue+skeleton+RNAlater slurry was stored in a -80ºC freezer until transport in a Doble-20 dry shipper (Chart MVE BioMedical, USA) aboard a commercial aircraft from Fiji to Taiwan under a permit (PVPS1300609) issued by the government of Fiji’s Ministry of Defence (sp.), National Security, and Immigration. Once at Taiwan’s National Museum of Marine Biology and Aquarium (NMMBA), the samples were centrifuged at 12,000 *xg* for 1 min to pellet the skeleton and tissues, and the RNALater was decanted. The tissue and skeleton were then re-suspended in 1 ml TRIzol® (Life Technologies), and RNA and DNA were extracted as described previously [2]. Proteins were left precipitated in acetone at -20ºC for future analysis.

**Nucleic acid extractions and real-time PCR**

RNAs were separated from DNAs and proteins with chloroform as recommended by the manufacturer, precipitated, purified with GeneMark Plant RNA spin columns (Taiwan) according to the manufacturer’s recommendations, dissolved in 30 μl DEPC-treated water, quantified on a Nanodrop spectrophotometer (Infinigen, Taiwan), and diluted to 20 ng μl^-1^. Similarly, DNAs were separated from the proteins via a back extraction buffer as described previously [3], precipitated, purified with Axygen’s PCR clean up columns (USA) according to the manufacturer’s recommendations, and re-suspended in 50 μl eluent provided by Axygen. DNAs were quantified as for RNA and diluted to 10 ng μl^-1^. Both RNAs and DNAs were electrophoresed on non-denaturing 1% Tris-borate-acetate (TBE)-agarose gels at 100 V for 30 min, stained in a bath of ethidium bromide for 15-20 min, and imaged under ultraviolet illumination with a digital camera (Olympus TG2, Japan) to assess nucleic acid quality.

The RNAs (10 μl, 200 ng) were converted to cDNA alongside an exogenous Solaris™ RNA spike (Life Technologies) with the High Capacity™ cDNA synthesis kit (20 μl reactions) as recommended by the manufacturer. Briefly, exogenous spikes are a preferred means of controlling for differences in the efficiency of the reverse transcription reaction [4] and circumvent the need for housekeeping genes, which cannot be validated with confidence in reef-building corals [3] due to their dual-compartmental nature (i.e., host+ dinoflagellate endosymbionts). cDNAs were diluted 4-fold in DEPC-treated water, and 2 μl were used in each real-time PCR (qPCR). Triplicate qPCRs were performed for each of the nine target genes and Solaris spike for a 70-sample subset of the ~100 samples whose RNAs and DNAs were extracted. For the Solaris spike assay, the manufacturer’s 20X primer/probe mix, which included a proprietary Taqman® (Life Technologies) FAM® probe, was used with appropriately diluted 2X Taqman real-time PCR mastermix with ROX® passive reference dye from Yeastern (Taiwan; 20 μl reactions). Thermocycling was performed on an Applied Biosystems 7500 real-time PCR machine (Life Technologies) at 95ºC for 10 min for 1 cycle followed by 40 cycles of 95ºC for 15 s and 60ºC for 60 s under the FAM setting as recommended by the manufacturer.

For the nine target genes (S2 table), a SYBR® Green-based mastermix (EZ-TIME™ real-time PCR mastermix w/ROX passive reference dye; Yeastern) was used (10 μl, 1X) with the primer concentrations and reaction conditions listed in the S2 table; specifics of the assay conditions are described in detail in prior works [5-8]. Inverse log threshold cycle values were normalized to recovery of the RNA spike and then divided by the host/*Symbiodinium* and *Symbiodinium*/host DNA ratios (i.e., host and *Symbiodinium* genome copy proportions [GCP], respectively) for the host coral and *Symbiodinium* target genes, respectively, to control for variable host/*Symbiodinium* biomass ratios between samples (see the main text for details.).

**Results A**

**Average live coral cover (ALCC)**

Coral cover averaged 33±13% (std. dev. for this and all error terms hereafter) across the 70 surveyed sites (S1 data file) and varied significantly across the eleven atolls/islands visited (1-way ANOVA, *p*<0.0001). This was similar to the ALCC of only the 46 sites from which corals were sampled (35±12%; Table 1). The island effect was driven by significantly lower ALCC at Vanua Balavu (20.4%) than at Totoya (35.6%), Cicia (38.4%), Nayau (43.0%), Fulaga (47.1%), and Mago (47.3%; Tukey’s honestly significant difference [HSD], *p*<0.05 for all five comparisons). ALCC also significantly differed across the three exposure categories (1-way ANOVA, *p*<0.01), due in part to significantly higher coral cover at reefs of intermediate exposure (36.2%) versus protected reefs (25.4%; Tukey’s HSD, *p*<0.05). ALCC was significantly higher (student’s *t*-test, *p*<0.01) on the fore reef (36.1%) than in the lagoon (25.4%). In contrast, ALCC did not differ significantly across the four reef types (1-way ANOVA, *p*=0.053). For the ALCC data from only the 46 sites from which corals were sampled, please see Table 1.

**Summary of physiological data**

Max. length and planar SA were determined for all 153 coral colonies from which biopsies were taken in Fiji’ Lau Archipelago, and RNAs and DNAs were extracted from a subset of 100 of these 153 samples. Of these 100 samples, 96 were genotyped, and the 13 molecular physiological response variables were assessed in a subset of 70 (the majority of which were genotyped; see S1 table.). Coral size (i.e., max. colony length and planar SA) is an important parameter for estimating reef age and health on an ecosystem-scale, but not on an organismal one; although large coral colonies may indeed display physiological differences from small ones, a large colony is not necessarily healthier or more “normal” than a small one. For this reason, the two size parameters were generally excluded from most MSA, especially those aimed at uncovering physiological outliers. The remaining 11 response variables included two biological composition parameters, the RNA/DNA ratio and the *Symbiodinium* GCP, and nine mRNAs (five and four from the coral host and *Symbiodinium*, respectively; discussed in the main text). In general, few of these parameters differed significantly across environment when tested via univariate ANOVAs (Table 2; described in detail below and in the main text).

**Island descriptions, host genotype breakdown, and outliers**

**Totoya**

The fore reefs were typically characterized by a diverse benthic community, with the scleractinian community differing by location. Shallower areas were dominated by branching acroporids and sub-massive poritids, with encrusting and plating corals also present. Small mounds of diverse, mixed-coral assemblages were also present at certain sites. At greater depths, colonies took on plating morphologies and formed series of shingle-like structures. Prominent macroalgae included *Halimeda* spp.*, Caulerpa* spp.*,* and *Dictyota* spp. Large colonies of *Diploastrea* sp. could be found at 30 m, and large tabular and branching acroporids were typical at 20-22 m. High coral cover, with large spur and groove ridges, was characteristic of the fore reefs, and the grooves were filled with both sediment and rubble. Shallower depths were dominated by small acroporid and pocilloporid colonies. *Lobophyton* sp. was common, as well.

Fore reef sand flat benthic cover was usually very low due to the mobile, largely unconsolidated nature of the substrate; this likely inhibited colonization by benthic organisms. However, large *Porites lobata* colonies and tabular acroporids were present within the sand flats at some sites, especially in deeper areas (30-50 m). Macroalgae became more common with depth due to the associated abatement of wave energy. The deeper fore reef habitats at the base of the reef were generally low relief sand flats that were mostly uncolonized. Often, there were sparse assemblages of cyanobacteria and small patches of macroalgae, along with isolated larger corals. In the lagoon (site FJTO06 only), the coral community composition was determined by the development stage of each patch. In the early stages, the coral community was dominated by massive species (e.g., *Porites* sp.) that tended to form pinnacles. Large *Diploastrea* sp. and poritid colonies served as the predominant framework, and acroporid colonies dominated the tops of the pinnacles.

Of the nine reef sites surveyed around Totoya (Fig 2a), pocilloporid corals were sampled from seven (Table 1). Of the 20 corals sampled, 13 were analyzed for the 13 molecular physiological response variables, and 12 of these 13 were genotyped; 9 (75%) and 3 (25%) were found to be *P. damicornis* and *P. meandrina*, respectively (Fig 1). Of the 13 samples analyzed, 2 were outliers, Totoya 7 and 15 (Fig 3d; Table 3; S1 and S3 tables); both *P. damicornis* samples were collected from the barrier fore reef, and both demonstrated aberrantly high *gfp-cp* mRNA expression levels (S3 table); the latter also demonstrated an aberrant host *lectin* mRNA expression level (S3 table).

**Matuku**

Fore reef sand flats were at the base of steep reef slopes at sites FJMT10-11 (Fig 2b). Visibility was poor, with a fair amount of suspended sediments present. Large branching acroporids with some recent mortality were common. *Drupella* sp. snails were found on several colonies, which could account for some of this recent mortality and scars on these acroporids. Plating *Pachyseris* spp. and *Echinopora* spp. colonies were common at the base of the slope. The lone lagoonal site surveyed (FJMT12) was located in the channel off a fringing reef system towards the west of the emergent landmass. Visibility was poor due to the proximity of the barren lagoon floor. Isolated coral colonies that are usually found in lagoonal environments were common. Large, monospecific stands of *Pavona clavus* and *Porites rus* covered almost 20 m of substrate in some areas. Several different leather corals were present, including *Lobophyton* sp. and *Sarcophyton* sp.

Of the five sites surveyed (Fig 2b), pocilloporid corals were sampled from three (Table 1). All 10 of the sampled colonies were genotyped and analyzed for all 13 molecular physiological response variables. Of these colonies, half were *P. verrucosa*, while 40 and 10% were *P. damicornis* and *P. meandrina*, respectively (Fig 1). Only one sample, Matuku 25 from site FJMT13, was considered an outlier (Fig 3d; Table 3; S1 and S3 tables), and this was largely due to an aberrantly high *rbcL* expression level in this *P. verrucosa* colony. Two samples, 22 and 27, had heat map scores of 1 but were not considered Mahalanobis distance outliers; the latter *P. damicornis* colony had an aberrantly high *gfp-cp* expression level (S3 table), and the former *P. verrucosa* colony had an aberrantly high *Symbiodinium* GCP (S1 table).

**Moala**

FJML19 and FJML20 were fore reefs located on the west side of the atoll (Fig 2c). Both sites were on the fore reef slope, with the slope and coral cover continuing beyond 40 m off the fore reef terrace. Upon the terrace, coral buildups on top of massive framework were common, with 15-20 m between such buildups. Acroporids and other branching corals were common on the tops of these buildups, which were characterized by moderate to high coral cover. Down the slope, *P. rus* plates were common, as were stands of staghorn acroporids. Feeding scars on table and plating corals were likely due to *Culcita* sp. sea stars, which were observed at high densities at these two sites.

Sites FJML17-18 were located near fore reef sand flats on the tops of shallow, fore reef terraces at the northwestern side of the atoll. On such terraces, a prominent spur and groove morphology was populated by small, branching coral colonies with old mortality colonized by CCA. *P. rus* colonies populated the edges of the overhangs where large sand flats were located, and oftentimes old coral rubble could be found in the vicinity; this could be evidence of a storm surge event. Most of the corals were smaller (~45-60 cm in diameter). At the terrace level, coral cover was lower, and CCA and turf algae cover were higher. On the east side of the atoll (FJML23, FJML15, FJML22, and FJML16), sites were on the fore reef slope. Large colonies of *Porites* spp. and *Diploastrea* sp. were common on the sides of the slope. Coming up the slope from 32 m, coral cover was low, but CCA were common. Small colonies of *Platygyra* spp., *Lobophyllia* spp., and *Echinopora* spp. were also common.

FJML21 was a lagoonal pinnacles site. The tops of the pinnacles, which rose from the lagoon floor, were coral-dominated and characterized by reef-derived debris in the sand (rubble-sized fractions). Pinnacles were usually circular in shape, but some were more elongated along hydrodynamic gradients. Coral cover was relatively high in the shallows (i.e., on the tops of the pinnacles). Tabular and branching acroporids were common. Specifically, *Echinophyllia* sp. and *Echinopora* spp. plates were common along the sides of the pinnacles. *Pachyseris rugosa* was also found. Along the sides of the pinnacles, the visibility was poor due to increased sediment loads. Finally, encrusting sponges and cyanobacteria were common at deeper depths in the lagoon.

Of the nine reef sites visited at Moala (Fig 2c), pocilloporid corals were sampled from six (Table 1). Of the 17 colonies sampled, 12 were analyzed for the molecular physiological response variables. Of these 12, 10 were genotyped (Fig 1); 80% were *P. verrucosa*, with the remaining two colonies being *P. meandrina* and *P. acuta*. Only sample 39, a *P. verrucosa* colony from site FJML18 (a leeward barrier fore reef) was considered an outlier (Fig 3d and Table 3), and this was due to an aberrant level of *lectin* mRNA expression in the host compartment (S3 table). Sample 45 had an aberrantly high RNA/DNA ratio, but this *P. verrucosa* colony was not considered a Mahalanobis distance outlier.

**Fulaga**

Two days were spent surveying reefs of Fulaga (Fig 2d), and six sites were visited (Table 1). Spur and groove assemblages were typical of the fore reef sites. Large, monospecific stands of foliaceous and plating *Pachyseris* spp.*, Leptoseris* spp.*, Montipora* spp., and *Merulina* spp. were present on more gradual slopes, while encrusting and laminar morphologies became more common at near-vertical angles. Fore reefs were typically gently sloping, with some scattered mounds and spurs of coral framework running perpendicular to the reef crest. A buildup of corals often forming a ridge or mound perforated by shore-perpendicular channels ran parallel to the reef crest in many areas. Large pinnacle reefs were found in the lagoon, and such lagoonal pinnacles had massive coral framework. The coral community composition was slightly different at each pinnacle, the tops of which were mostly coral-dominated; pocilloporids, acroporids, and stylophorids were all abundant. Surrounding the pinnacles were sediment-dominated lagoon floors. Few colonized coral communities (usually branching/tabular *Acropora* spp.) were common in these generally low-relief areas, which were characterized by large grain sediment deposits.

Of the four sites from which pocilloporid corals were sampled (Table 1), seven of the nine colonies sampled were analyzed for molecular physiological response variables, and eight of the nine were genotyped (Fig 1); half of the genotyped colonies were *P. damicornis*, with the other half being *P. acuta*. Of the four *P. acuta*, all were analyzed for molecular physiological response variables, and one was considered an outlier (Fig 3d and Table 3); sample 56 had a heat map score of 4 due to aberrant *Symbiodinium rbcL*, *hsp90*, and *ubiq-lig*, and aberrant host coral *cu-zn-sod* mRNA expression levels (S3 table). This was the highest heat map score in the dataset, and PCA (discussed in the main text; Fig 3b) also revealed this sample to be an outlier. One other sample, 54, was likely an outlier, as well, due to aberrant *Symbiodinium rbcL*, *ubiq-lig*, and *apx1* mRNA levels (S3 table); however, due to the low DNA extraction efficiency, the RNA/DNA ratio, and therefore the Mahalanobis distance, could not be calculated with confidence. Nevertheless, see the treatise on re-assigning this coral sample as an outlier using imputed data in the Discussion of the main text. Both samples 54 and 56 were from very shallow (2.4 m) depths within a lagoonal patch reef characterized by incredibly high sedimentation/turbidity levels (visibility=1 m or less). The final sample from the same site, 55, showed aberrant expression levels for the host *cu-zn-sod* mRNA but was not considered to be an outlier (Mahalanobis distance < 4.29).

**Kabara**

Five sites were surveyed over the course of two days at Kabara (Fig 2e and Table 1). Fore reef sites (FJKA30 and FJKA32-34) had spur and groove morphologies on the terrace (5-12 m), with large buildups of sediment in between spurs. At ~ 15 m the slope became very steep, with numerous, small- to medium-sized acroporids and pocilloporids found in abundance. CCA cover was high, and there was little macroalgae. Small colonies of encrusting corals were common. FJKA31, a pinnacles site, had mixed habitats: 1) shallow, fore reef terraces with small acroporid colonies and 2) fore reef sand flats. Large *P. lobata* colonies and tabular acroporids occurred within the sand flats, especially in deeper areas (30-50 m).

No lagoonal sites were visited at Kabara, and of the 13 colonies sampled across two of the five surveyed fore reef sites (Table 1), only six were genotyped (Fig 1); all were found to be *P. acuta*. Four of these six genotyped samples, all of which were from the leeward pinnacles site, were analyzed for the 13 molecular physiological response variables, and one (sample 68), was found to be an outlier (Table 3 and Fig 3d) that demonstrated aberrant expression levels of the following three gene mRNAs (i.e., heat map score=3): *Symbiodinium hsp90* and *ubiq-lig* and host coral *cu-zn-sod* (S3 table). None of the other three samples from that same site (FJKB31) exhibited aberrant levels of any of the response variables.

**Vanua Vatu**

Only one day was spent surveying reefs of Vanua Vatu (Table 1), and three fore reef sites were surveyed; sites FJVV36, FJVV37, and FJVV35 were all located on the west side of the atoll, at which large colonies of massive *Porites* spp. were intermixed with *Halimeda* sp. clumps. At ~ 15 m, typical spur and groove structures were evident. CCA and smaller acroporids were common in the shallows. *Peyssonnelia* sp. was common at all depths. Specifically at site FJVV37, large plates of *Pachyseris* spp. and *Merulina* spp. were common. Overall coral cover was high at Vanua Vatu (41%; see the S1 data file.), namely because of the abundance of 1) large colonies of *Porites* spp. and 2) numerous plating corals. No lagoonal sites were visited. Six pocilloporid corals were sampled across the three sites, and, of the three genotyped, two and one were *P. verrucosa* and *P. meandrina*, respectively (Fig 1). Due to the small sample size, none of the three genotyped samples were analyzed further for the molecular physiological response variables.

**Nayau**

One day was spent surveying reefs of Nayau (Table 1), and all fore reef sites (FJNA38-40) were located on the northwest side of the atoll on a shallow, fore reef slope. Large buildups of acroporids comprised the majority of the reef morphologies. At the base of these reef buildups were large piles of rubble. Macroalgae (e.g., *Tydemania expeditionis*) were also common on the sides of the reef buildups. *Drupella* sp. snails were found on several *Acropora* spp. colonies. An old anchor chain at site FJNA38 was colonized by small *Pocillopora* sp. colonies and encrusting sponges. Large plating/sheeting colonies of *Diploastrea* sp. were prominent at 15 m. FJNA39 was characterized by a classic spur and groove morphology, with higher coral cover on the tops of the spurs. The grooves were scoured, with large pieces of coral rubble and sediment present. No sites were visited in the lagoon, and pocilloporid corals were sampled from only one of the three fore reef sites: FJNA38. Of the seven corals sampled from this leeward barrier fore reef, six were genotyped, and all were found to be *P. verrucosa* (Fig 1). Due to the small sample size, none of these genotyped samples were analyzed further for the molecular physiological response variables.

**Tuvuca**

Three reef sites were surveyed around Tuvuca (Fig 2f), where one day was spent (Table 1). FJTV41 was a fore reef located on the northwest side of the atoll. At the deep end of the slope there were large colonies of *P. rus*. Isolated, monospecific *Isopora* spp. stands were found around 16 m. At FJTV43 there was notably more mortality on tabular acroporids than at the other two fore reef sites. Some of the smaller acroporids had old mortality that had been colonized by algae, and large *Diploastrea* sp. colonies had old mortality that had been colonized by *Peyssonnelia* sp. As was common at many other sites in Lau Province, shallow, fore reef slopes had coral assemblages of predominantly pocilloporids, stylophorids, and acroporids.

No lagoon sites were visited, and eight pocilloporid corals were sampled across two of the three surveyed sites; all eight were both genotyped and assessed for the 13 molecular physiological response variables. Regarding the genotype breakdown (Fig 1), five and three colonies were found to be *P. verrucosa* and *P. acuta*, respectively. No outliers were identified amongst the eight samples (Table 3); however, sample 88 displayed an aberrant level of the *Symbiodinium* GCP (S1 table); 67% of the extracted DNA was from the *in hospite* *Symbiodinium* populations, suggesting that these dinoflagellates were overgrowing the coral tissues. Sample 87, which was from the same site (FJTV41; a leeward barrier reef) displayed an aberrant expression level of host coral *lectin* (S3 table).

**Cicia**

Five sites were surveyed over the course of two days around Cicia (Fig 2g and Table 1). At the fore reef sites, the benthic community was highly diverse and coral-dominated, often with a substantial macroalgal component. Environmental and disturbance regimes appeared to have governed the benthic community structure. Scleractinian diversity was very high and was often dominated by acroporids, pocilloporids, poritids, and favids. The edge of the shallow slope was typically dominated by large *Pocillopora eydouxi* colonies, thick-branched and elkhorn-like *Isopora* sp*.,* and unusually large *Favia stelligera* and/or *Pavona maldivensis* stands. The shallower areas of the reef flat were exposed at low tide and dominated by CCA. During the visit, 212 crown of thorns sea stars (COTS) were removed, as an active outbreak was occurring.

No lagoon sites were visited, and 12 pocilloporid corals were sampled from three of the five fore reef sites. Six of these corals were genotyped; five and one were found to be *P. verrucosa* and *P. damicornis*, respectively (Fig 1). Of the 12 sampled colonies, 8 (including the 6 genotyped colonies) were assessed for the 13 response variables. No outliers were identified; however, sample 93 from site FJCC44 showed an aberrantly high expression level of the host coral *ca* mRNA (S3 table). Strangely, sample 99, which was from the same site, was from the healthy portion of a colony that was estimated to be 5-10% bleached and demonstrated an aberrantly high RNA/DNA ratio (S1 table); a bleached portion of this same colony (sample 98), on the other hand, did not display aberrant levels of any of the response variables.

**Mago**

Three reefs were surveyed at Mago (Fig 2h) over the course of one day (Table 1) after obtaining permission from owner Mel Gibson. Fore reef sites were characterized by spur and groove assemblages, with steep slopes dominated by 1) large, isolated colonies of *P. lobata* and 2) tabular acroporids. Some COTS predation (large swaths of white corals) was evident. Clumping *Halimeda* sp. algae were found interspersed among branching corals. Large, sheeting colonies and plating corals were common along the steep slopes. Several large, table acroporids had been turned over during a recent storm. CCA were common and covered most of the old, dead corals on the reef terrace. *Echinophyllia* sp*.* colonies were common around 30 m at the base of the steep slope. No lagoon sites were visited.

Nine pocilloporid corals were sampled across the three sites; all of which were genotyped (Fig 1). Unlike other islands visited, a broad diversity of pocilloporids was sampled: 3 *P. meandrina*, 3. *P. acuta*, 1 *P. damicornis,* 1 *P. verrucosa*, and 1 *P. brevicornis*. The latter was the only *P. brevicornis* colony identified on the mission. All nine genotyped colonies were assessed for the 13 response variables, and one colony (sample 111) was found to be an outlier (Fig 3d and Table 3). This *P. acuta* sample was from a leeward fringing fore reef and exhibited an aberrant expression level of *Symbiodinium ubiq-lig* only (S3 table).

**Vanua Balavu**

The last region of Fiji’s Lau Province surveyed was the Vanua Balavu Archipelago (Fig 2i), and 19 sites were surveyed over the course of five days (Table 1). In terms of the fore reef sites, those on the east side of the atoll (FJVB52-55) were located on the shallow and deep fore reef slopes. Reef slopes had small- to medium- sized acroporids. At the base of the slope were large clumps of cyanobacteria. Isolated, large colonies of *P. lobata* were present around 35 m, and plating *P. rus* colonies occupied nearly 100% of the benthos at 35 m in certain areas. Large gorgonians and soft corals were common on the slope. *Caulerpa* sp. was also more common along the shallower portion of the reef slope. Higher densities of sea cucumbers were present at the fore reef sites that at the other 10 islands/atolls surveyed in Lau Province. *Millepora* spp. were also more common than at previous areas visited on the expedition. Some COTS predation was noted at FJVB55, where large plates of *Isopora* spp. were common.

Sites FJVB57, FJVB60-61, and FJVB69 were located on the fore reef slope. Large accumulations of green macroalgae and cyanobacteria were found at the base of these slopes. The fore reef terrace and slope had low coral cover; leather corals and *Millepora* spp. were found in this area. Large mats of cyanobacteria and old dead coral were both commonly observed. At site FJVB61, *Diploastrea* sp. colonies at 20 m had evidently been infected by trematodes. Diseased poritid colonies were also common. One *P. clavus* colony that was at least 10 m in diameter was documented.

The lagoonal sites FJVB58-59, FJVB62-65, and FJVB70 were all pinnacle reefs; these pinnacles were massive framework buildups, with branching and tabular corals on the tops and sides. *Halimeda* sp. was common in patches. Common coral species included *Turbinaria peltata, Galaxea* sp., *Pachyseris rugosa,* and *Hydnophora rigida*. Both free-living corals and motile invertebrates were relatively common compared to islands/atolls visited earlier in the mission. Lagoonal sand flats were extensive and surrounded each pinnacle. At site FJVB70, several large colonies of *P. lobata* and *Diploastrea* sp. had extensive mortality.

FJVB67 was a lagoonal fringing reef in which large patches of *Cladophora* sp. were common at 30 m. Large, plating *Diploastrea* sp. colonies were recorded at 30 m, as were large colonies of branching acroporids. Some feeding scars were noticed on these branching acroporids, possibly due to COTS. Soft corals were also common on the lagoonal fringing reefs. FJVB68 was located in the northwestern corner of the lagoon and was comprised of a series of lagoonal patch reefs. Coral community composition was composed of dense *Acropora* spp. thickets.

Of the 42 corals sampled across 12 of the 19 sites surveyed, 18 and 19 were genotyped and assessed for the 13 response variables, respectively (18 of the 19 latter samples represented the genotyped samples.). Of the 18 samples genotyped (Fig 1), 3 and 15 were *P. damicornis* and *P. acuta*, respectively. One of the three *P. damicornis* samples was an outlier (Table 3 and Fig 3d); sample 115 from a leeward barrier fore reef displayed an aberrant expression level of the *Symbiodinium* *zifl1l* mRNA (S3 table). Of the 15 *P. acuta* samples, 3 were outliers (Table 3): samples 123 (FJVB55), 146 (FJVB63), and 153 (FJVB67). The former sample was from a barrier fore reef and displayed aberrant expression levels of host coral *ca* and *cu-zn-sod* (S3 table). Sample 146 was from a lagoonal patch reef and displayed aberrant expression levels of *Symbiodinium hsp90* and *ubiq-lig* (S3 table). Sample 153 was from a windward fringing reef and displayed aberrant expression levels of *Symbiodinium rbcL* and host coral *ca* (S3 table).

**Multivariate ANOVA (MANOVA)**

When using MANOVA (Table 2), only one environmental parameter, sampling date, had a significant effect on the multivariate centroid encompassing all 13 molecular physiological response variables when using the Bonferroni-adjusted α of 0.004. Although sampling date was not hypothesized to affect coral physiology in a biologically meaningful manner, one date, June 4, 2013, was fairly well separated from all others along CA1 and 2; the former axis was characterized by a negative relationship between *Symbiodinium rbcL* and *ubiq-lig* mRNA expression, with the latter by a negative correlation between max. length and planar SA (data not shown).

**Univariate and multivariate statistics by species**

When looking at univariate effects of the environmental parameters on the molecular physiological response variables for the three species for which greater than 15 samples were analyzed-*P. damicornis*, *P. acuta*, and *P. verrucosa*- only three Bonferroni-adjusted significant differences were uncovered (S4 table). First, *zifl1l* mRNA expression varied significantly over sampling time and ALCC for *Symbiodinium* populations within *P. verrucosa* only. Regarding the former difference, expression of this gene was nearly 50-fold reduced in samples collected after 14:00 compared to those sampled prior to this time (Tukey’s HSD, *p*<0.05 vs. <10:00 and 10:00-14:00). Regarding the ALCC difference, there were too few samples at certain ALCC groupings to interpret this difference with confidence. The max. length of *P. verrucosa* colonies also varied significantly across colony depths; colonies found between 5 and 10 m were significantly smaller than those sampled <5 m and at 10-15 m. Finally, the multivariate centroid of *P. acuta* varied across several environmental parameters at an α level of 0.05, including island, exposure, reef zone, reef type, and ALCC, and it could be argued that MANOVA does not necessitate the Bonferroni adjustment since all response variables are analyzed simultaneously. Therefore, these MANOVA-documented differences for *P. acuta* may shed light on the environmental physiology of this species and warrant future investigation

**S1 references**

1. Saul S, Purkis S. Semi-automated object-based classification of coral reef habitat using discrete choice models. Remote Sensing*.* 2015;7: 15894-15916.
2. Mayfield AB, Bruckner AW, Chen CH, Chen CS. A survey of pocilloporids and their endosymbiotic dinoflagellate communities in the Austral and Cook Islands of the South Pacific. Platax. 2015;12: 1-17.
3. Mayfield AB, Hirst MD, Gates RD. Gene expression normalization in a dual-compartment system: a real-time PCR protocol for symbiotic anthozoans. Mol Ecol Res. 2009;9: 462-470.
4. Bower NI, Moser RJ, Hill JR, Lehnert SA. Universal reference method for real-time PCR gene expression analysis of preimplantation embryos. Biotechniques. 2007; 42: 199-206.
5. Mayfield AB, Chen CS, Liu PJ. Decreased green fluorescent protein-like chromoprotein gene expression in specimens of the reef-building coral *Pocillopora damicornis* undergoing high temperature-induced bleaching. Platax. 2014;11: 1-23.
6. Mayfield AB, Chen YY, Dai CF, Chen CS. The effects of temperature on gene expression in the Indo-Pacific reef-building coral *Seriatopora hystrix*: insight from aquarium studies in Southern Taiwan. Int J Mar Sci. 2014;4(50): 1-23.
7. Mayfield AB, Hsiao YY, Chen HK, Chen CS. Rubisco expression in the dinoflagellate *Symbiodinium* sp. is influenced by both photoperiod and endosymbiotic lifestyle. Mar Biotech. 2014;16: 371-384.
8. Mayfield AB, Wang YB, Chen CS, Chen SH, Lin CY. Compartment-specific transcriptomics in a reef-building coral exposed to elevated temperatures. Mol Ecol. 2014; 23: 5816-5830.
9. Mayfield AB, Chan PS, Putnam HM, Chen CS, Fan TY. The effects of a variable temperature regime on the physiology of the reef-building coral *Seriatopora hystrix*: results from a laboratory-based reciprocal transplant. J Exp Biol. 2012;215: 4183-4195.
10. Mayfield AB, Fan TY, Chen CS. Real-time PCR-based gene expression analysis in the model reef-building coral *Pocillopora damicornis*: insight from a salinity stress study. Platax. 2013;10: 1-29.
11. Mayfield AB, Chen CS, Dempsey AC, Bruckner AW. The molecular ecophysiology of closely related pocilloporids from the South Pacific: a case study from the Austral and Cook Islands. Platax. 2016;13: 1-25
